# Dual‐Function Cosubstrates Enable Threonine Dehydrogenase‐Driven Chemoenzymatic Pyrrole Synthesis

**DOI:** 10.1002/anie.6159381

**Published:** 2026-10-01

**Authors:** Valentina Jurkaš, Fabian M. Kulier, Jorge González‐Rodríguez, Carlotta Chiesa, Peter Both, Peter Šiška, Fabio Parmeggiani, Florian Rudroff, Margit Winkler

**Affiliations:** ^1^ Austrian Centre of Industrial Biotechnology (ACIB) Graz Austria; ^2^ Institute of Molecular Biotechnology Graz University of Technology Graz Austria; ^3^ Institute of Applied Synthetic Chemistry TU Wien Vienna Austria; ^4^ Politecnico di Milano, Dipartimento Di Chimica Materiali ed Ingegneria Chimica “Giulio Natta” Milan Italy; ^5^ Axxence Slovakia s.r.o. Bratislava Slovakia; ^6^ CCBio Institute of Chemistry and Biotechnology ZHAW Zurich University of Applied Sciences Wädenswil Switzerland

**Keywords:** biotransformation, chemoenzymatic Knorr pyrrole synthesis, cofactor recycling, dual‐function cosubstrate, threonine dehydrogenase

## Abstract

Preparative biocatalytic synthesis of substituted pyrroles remains challenging despite recent advances in enzymatic α‐aminoketone generation. In this study, we report a concurrent chemoenzymatic cascade to disubstituted pyrroles based on threonine dehydrogenase (ThrDH)‐catalyzed generation of aminoacetone from l‐threonine that is intercepted in situ by Knorr pyrrole condensation with *β*‐dicarbonyl compounds. To overcome cofactor limitations at high substrate concentrations, *Escherichia coli* ThrDH was coupled to an alcohol dehydrogenase from *Rhodococcus ruber* DSM 44541 (ADH‐A), enabling dual‐function of the *β*‐dicarbonyl cosubstrate as both a hydride acceptor and pyrrole building‐block. The whole‐cell catalyst tolerated up to 8% (v/v) *β*‐dicarbonyl and afforded pyrrole products in up to 90% yield and space‐time yields of 4.5 g L^−1^ h^−1^. Preparative synthesis on a 100 mL scale furnished 4.3 g of pyrrole (279 mM, 93% isolated yield). Among the products obtained, the cascade provides direct access to a reported sunitinib intermediate, illustrating its potential utility for pharmaceutical synthesis. This work establishes ThrDHs as outstanding biocatalysts for heterocycle synthesis and demonstrates that amino acid feedstocks can be efficiently converted into substituted pyrroles through a scalable chemoenzymatic cascade.

## Graphical abstract

Classical Knorr chemistry meets contemporary biocatalysis. Threonine dehydrogenase generates aminoacetone from l‐threonine, which is directly intercepted in a chemoenzymatic Knorr pyrrole synthesis. A dual‐function *β*‐dicarbonyl substrate serves as both pyrrole precursor and hydride acceptor for integrated NAD^+^ recycling, affording substituted pyrroles in high titers.

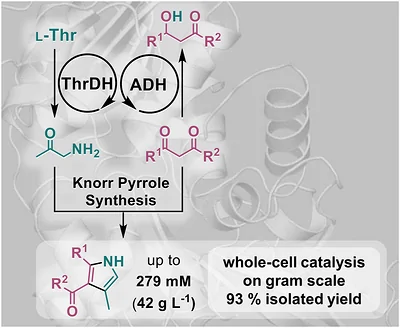

Pyrroles are privileged *N*‐heterocycles found in pharmaceuticals, natural products, and functional materials [1, 2, 3]. Among the numerous methods available for pyrrole synthesis, the classical Knorr pyrrole synthesis [4] (KPS) remains a versatile approach to substituted pyrroles based on the condensation of α‐aminoketones with *β*‐dicarbonyl compounds [5]. The propensity of α‐aminoketones for self‐condensation has motivated numerous strategies for their in situ preparation [6, 7, 8, 9]. Recent chemical and biocatalytic approaches have explored the in situ generation of α‐aminoketones, including biocatalytic methods based on pyridoxal phosphate‐dependent enzymes such as transaminases and α‐oxoamine synthases. In the reported transaminase‐based approaches, α‐aminoketone formation has often been demonstrated with substrates bearing aromatic substituents, which can contribute to productive substrate positioning and/or stabilization of the resulting intermediates [10, 11]. α‐Oxoamine synthases provide an alternative route to aliphatic α‐aminoketones, but their application can involve elevated temperatures and multiple cofactors [12, 13, 14]. KPS‐independent chemoenzymatic pyrrole syntheses have also been reported through other enzymatic strategies, including enzymatic generation of pyrrole precursors from d‐glucarate and oxidative aromatization of 3‐pyrrolines [15, 16]. Threonine dehydrogenases (ThrDHs) offer a direct route from an amino acid to an α‐aminoketone, catalyzing the oxidation of l‐threonine (l‐Thr) to 2‐amino‐3‐ketobutyrate, which spontaneously decarboxylates to aminoacetone. Previous chemoenzymatic cascades deliberately exploited the spontaneous self‐condensation of ThrDH‐generated aminoacetone to afford pyrazines [17, 18, 19, 20, 21]. In contrast, we envisioned diverting the reactive aminoacetone intermediate away from self‐condensation into a concurrent Knorr pyrrole synthesis through rapid interception with *β*‐dicarbonyl compounds, thereby establishing a concise one‐pot chemoenzymatic route to substituted pyrroles under mild conditions.

Because ThrDH‐catalyzed l‐Thr oxidation generates NADH, we hypothesized that the *β*‐dicarbonyl cosubstrate could simultaneously function as a pyrrole precursor and a hydride acceptor for alcohol dehydrogenase‐mediated NAD^+^ regeneration, enabling integrated cofactor recycling through a dual‐function cosubstrate strategy (Scheme 1).

**SCHEME 1:**
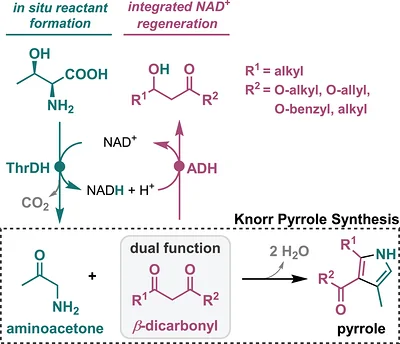
Concept of the ThrDH‐driven chemoenzymatic pyrrole synthesis. ThrDH generates aminoacetone from l‐Thr, enabling in situ Knorr pyrrole synthesis (KPS) under mild conditions. Coupling to an ADH enables integrated NAD^+^ regeneration through a dual‐function cosubstrate strategy, in which the *β*‐dicarbonyl acts both as a pyrrole precursor and a hydride acceptor.

ThrDHs are ubiquitous enzymes involved in l‐Thr biodegradation pathways [22]. They belong to two distinct superfamilies of alcohol dehydrogenases (ADHs): the cofactor‐free short chain dehydrogenase/reductase (SDR) and Zn‐dependent medium‐chain dehydrogenase/reductase (MDR) family [23]. We chose the *Cn*ThrDH from *Cupriavidus necator* [18, 24, 25, 26] and *Ec*ThrDH from *Escherichia coli* [19, 27, 28, 29, 30, 31] as SDR and MDR representatives, respectively. In addition, we investigated *Bs*ThrDH from *Bacillus subtilis* [32, 33, 34] and *Lok*ThrDH from *Loktanella* sp. [35] as new homologs of the *Cn*ThrDH and *Ec*ThrDH, selected from organisms in which ThrDH‐associated metabolites have been detected.

Even though the SDR‐type enzymes exhibited an order of magnitude higher catalytic efficiencies in initial‐rate assays (Figure 1B) as well as better pyrrole **2a** production at low substrate loading, *Ec*ThrDH performed best at elevated l‐Thr concentrations (Figure 1C), suggesting better operational stability under process conditions and/or reduced susceptibility to inhibition by pathway intermediates.

**FIGURE 1:**
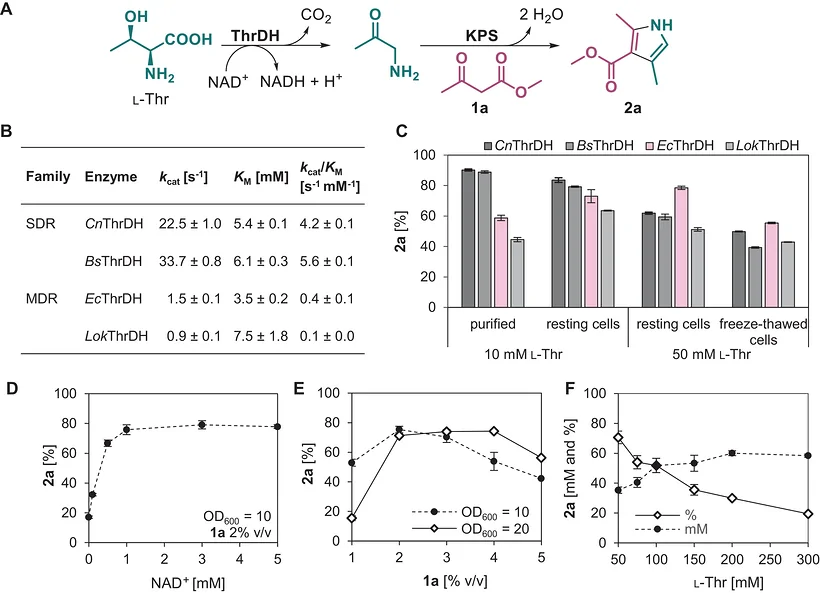
Characterization of the ThrDH‐driven chemoenzymatic cascade for pyrrole **2a** synthesis. (A) One‐pot chemoenzymatic pyrrole synthesis using threonine dehydrogenase (ThrDH) for generation of aminoacetone, followed by in situ Knorr pyrrole synthesis (KPS) with methyl acetoacetate (**1a**). (B) Kinetic characterization of ThrDH homologues. Spectrophotometric initial rate NADH formation in the presence of l‐Thr. (C) Screening of different ThrDH homologues and catalyst formats under varying reaction conditions. Reactions were performed with 3% (v/v) **1a** and either purified ThrDH (1 mg mL^−1^) and 10 mM of NAD^+^ or whole cells (OD_600_ = 10) and 1 mM of NAD^+^ for 18 h at 37°C. Effect of (D) NAD^+^ concentration, (E) **1a** loading and (F) l‐Thr concentration on pyrrole **2a** formation using *Ec*ThrDH resting cells. Unless stated otherwise, reactions included cells at OD_600_ = 20, 1 mM NAD^+^, 50 mM l‐Thr, and **1a** at 4% (v/v) in KPi 100 mM, pH 7.5, incubated 18 h at 37°C. Values represent the mean ± standard deviation of technical triplicates; detailed conditions are found in the Supporting Information.

As the whole‐cell reactions relied on the intrinsic capacity of *E. coli* metabolism to regenerate the NAD^+^, cells subjected to a freeze‐thaw cycle were less productive than the metabolically active resting cells. The reaction was further characterized in terms of **1a**, NAD^+^ and l‐Thr loading. Product formation strongly depended on exogenous NAD^+^, indicating that the endogenous intracellular cofactor pool was insufficient to sustain efficient turnover by the overexpressed *Ec*ThrDH. Product titers increased sharply up to 1 mM NAD^+^, beyond which no substantial improvement was observed (Figure 1D). The maximum tolerated **1a** concentration before a decline in product formation was 2% (v/v) at OD_600_ = 10 and 4% (v/v) at OD_600_ = 20 (Figure 1E). Increasing the l‐Thr concentration identified an apparent plateau in **2a** formation at approximately 60 mM (Figure 1F). The plateau indicated that oxidation of l‐Thr became limited by intracellular NAD^+^ availability.

Since ThrDH activity continuously generates NADH, we hypothesized that product formation could be improved by coupling the cascade to an auxiliary NADH‐consuming enzyme. Reduction of *β*‐keto esters is known to generate thermodynamically favored *β*‐hydroxy esters capable of intramolecular hydrogen‐bond stabilization, thereby providing a thermodynamic driving force for substrate‐coupled NAD^+^ recycling through a “smart cosubstrate” concept [36, 37, 38]. Only trace amounts of the reduction product **3a** were detected in reactions containing *Ec*ThrDH resting cells alone (Figure S9), indicating a lack of suitable alcohol dehydrogenase (ADH) in *E. coli* background. Coupling the cascade to ADH from *Rhodococcus ruber* DSM 44541 (ADH‐A) [39, 40, 41, 42] indeed enabled cosubstrate **1a** to serve a dual function as both pyrrole precursor and hydride acceptor for much more efficient aminoacetone generation (Figure 2A).

**FIGURE 2:**
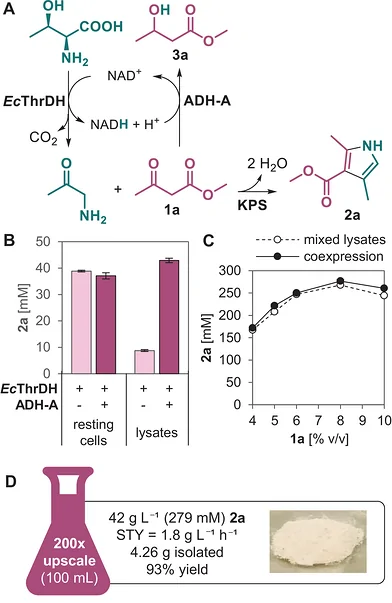
Implementation of ADH‐A catalyzed NAD^+^ regeneration using **1a** as a dual‐function cosubstrate. (A) Coupling of ADH‐A to the ThrDH‐driven cascade for pyrrole **2a** synthesis, in which **1a** serves as both the pyrrole precursor and a “smart” cosubstrate for in situ NAD^+^ regeneration. (B) Comparison of different ADH‐A implementation strategies, including addition as a second whole‐cell catalyst or use of both enzymes as lysates at 50 mM l‐Thr and 4% v/v **1a**. (C) Effect of **1a** loading on **2a** formation at 300 mM l‐Thr using mixed *Ec*ThrDH/ADH‐A lysates or a freeze‐thawed coexpression strain. (D) Preparative synthesis of **2a** on 100 mL scale using the coexpression strain [OD_600_ = 20, l‐Thr 300 mM, **1a** 8% v/v (2.4 eq), NAD^+^ 2 mM (0.67 mol%)]. Error bars correspond to standard deviations of technical triplicates. STY, space‐time yield.

While combining separate resting‐cell catalysts yielded no improvement over *Ec*ThrDH alone, cell lysis markedly reduced pyrrole formation by *Ec*ThrDH. Supplementation with ADH‐A restored product formation, consistent with the loss of intracellular cofactor recycling and reduced mass‐transfer barriers in the lysate (Figure 2B). Corresponding amounts of the reduction product **3a** were detected (Figure S10). Coexpression of *Ec*ThrDH and ADH‐A in a single strain reproduced the performance of mixed lysates while simplifying catalyst preparation and maintaining catalytic activity after freeze‐thaw treatment (Figure S11B). Both mixed lysates and the coexpression strain tolerated up to 8% (v/v) **1a**, overcoming the ∼60 mM product plateau observed with *Ec*ThrDH alone and producing up to 270 mM (90%) of **2a** from 300 mM l‐Thr (Figure 2C).

Despite the known reactivity of aminoacetone, oxidative dimerization side‐product 2,5‐dimethylpyrazine [7, 11, 21] was detected only in minor amounts under optimized conditions, indicating efficient interception by the Knorr condensation step (see Section S2.3). We further investigated whether aminoacetone may serve as a substrate for ADH‐A. LC‐MS analysis of benzoylated reaction samples detected aminoacetone and 1‐aminopropan‐2‐ol under optimized conditions. 1‐Aminopropan‐2‐ol was also detected in the absence of ADH‐A, indicating endogenous *E. coli* activity (Figure S40). Conversely, 1‐aminopropan‐2‐ol afforded pyrrole **2a** in the absence of l‐Thr, indicating that the aminoacetone/1‐aminopropan‐2‐ol interconversion is reversible under the applied conditions (Figure S18).

To demonstrate the preparative applicability of the developed chemoenzymatic cascade, the reaction was performed on a 100 mL scale (200‐fold volume increase) under optimized conditions using 300 mM l‐Thr and 8% (v/v) **1a** (Figure 2D). Analytical‐scale time‐course experiments revealed rapid product formation, with space‐time yields (STY) of 6.8 g L^−1^ h^−1^ after 2 h and 4.5 g L^−1^ h^−1^ after 8 h (Figure S12). Due to the low aqueous solubility of **2a** and its precipitation during the reaction, representative monitoring of the preparative‐scale transformation was not feasible. The reaction was therefore continued for 24 h to maximize overall conversion and product recovery. Then, 4.26 g of **2a** were isolated, corresponding to an isolated yield of 93% and STY of 1.8 g L^−1^ h^−1^. For comparison, the largest isolated product amount reported for a biocatalytic pyrrole synthesis to date is 93 mg for a structurally distinct pyrrole [10], highlighting the preparative potential of the present cascade.

Exploring the product scope (Figure 3) showed that methyl and ethyl esters afforded up to 90% analytical yield, while increasing steric bulk at either the ester or ketone substituent progressively reduced conversion. Acetylacetone **1g** gave **2g** in good yields, demonstrating that ester functionality is not essential for turnover. In contrast, cyclic cosubstrates afforded only trace reactivity. Direct reaction with aminoacetone likewise resulted in very low pyrrole formation, indicating that the Knorr condensation represents a major bottleneck for these substrates under the investigated conditions (Figure S15). These results demonstrate excellent performance for small linear *β*‐keto esters with steric bulk progressively limiting conversion.

**FIGURE 3:**
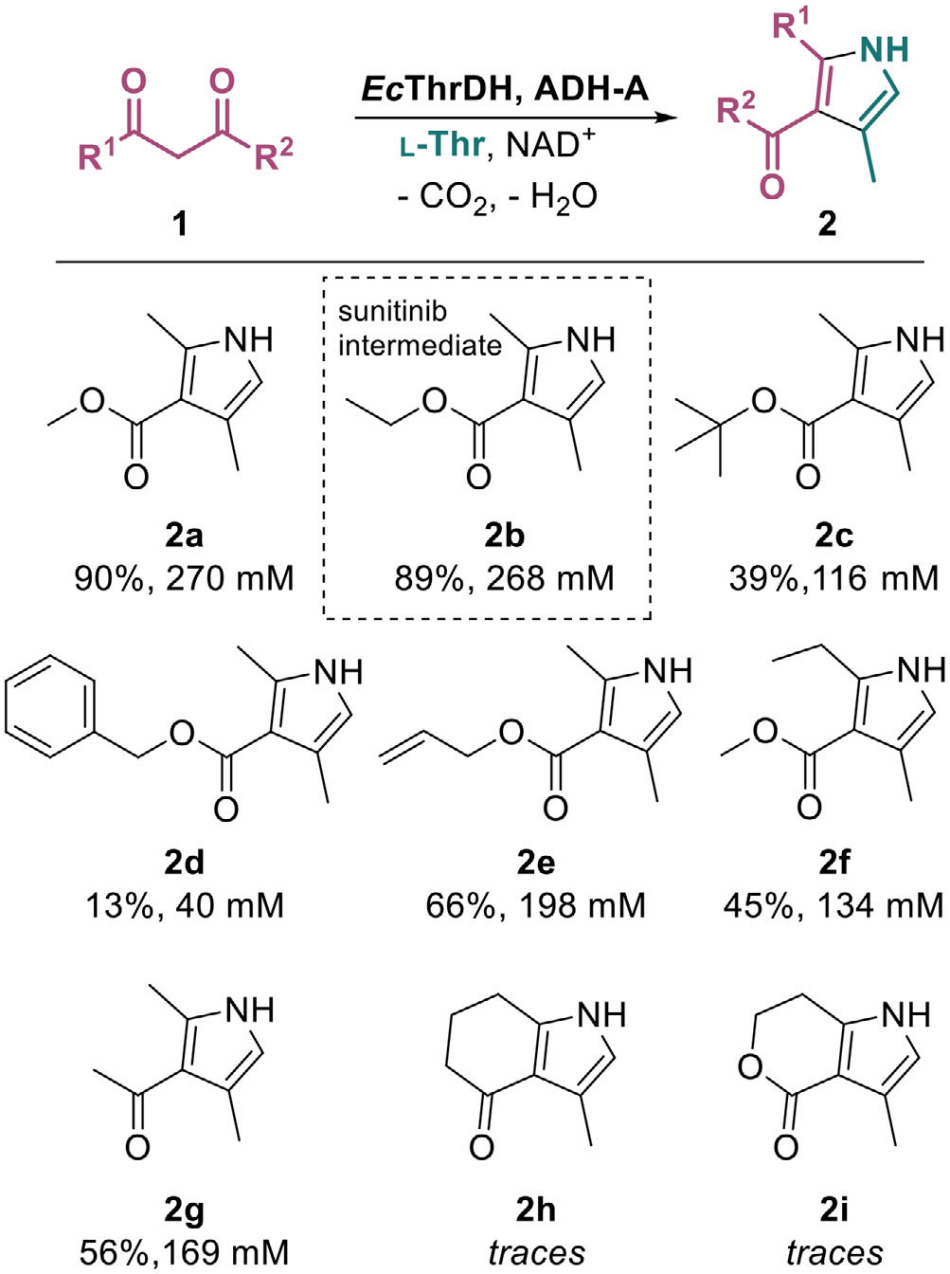
Formation of disubstituted pyrroles with alternative *β*‐dicarbonyl cosubstrates. Reaction conditions: freeze‐thawed *E. coli* cells coexpressing *Ec*ThrDH and ADH‐A (OD_600_ = 20), l‐Thr (300 mM, 1 equiv.), *β*‐dicarbonyl **1a–f** [8% (v/v), corresponding to 2.43, 2.09, 1.56, 1.51, 1.85, and 2.09 equiv., respectively], **1g** (4% (v/v), 1.29 equiv.), or **1h,i** (100 mM, 0.33 equiv.), NAD^+^ (1 mM, 0.33 mol%) in KPi (100 mM, pH 7.5). Incubated 18 h at 37°C and 130 rpm. Traces (≤ 5% peak area relative to **2a**).

Notably, pyrrole **2b** is a reported synthetic intermediate in the preparation of the tyrosine kinase inhibitor sunitinib, an anticancer drug used for the treatment of renal cell carcinoma and gastrointestinal stromal tumors [43]. Its direct formation from l‐Thr demonstrates how the developed cascade can furnish pharmaceutically relevant pyrrole intermediates from simple amino acid feedstocks under mild conditions.

To our knowledge, this is the first chemoenzymatic pyrrole synthesis based on ThrDH‐catalyzed aminoacetone generation from l‐Thr. Coupling the cascade to ADH‐A enabled integrated NAD^+^ regeneration through a dual‐function cosubstrate strategy, in which the *β*‐dicarbonyl serves simultaneously as pyrrole precursor and hydride acceptor. The system operates in whole‐cell format and tolerates substrate concentrations rarely observed in biotransformations with non‐engineered enzymes. Substituted pyrroles were produced in high yields, including gram‐scale synthesis with 93% isolated yield. The cascade achieved pyrrole titers of up to 42 g L^−1^, demonstrating synthetically relevant productivity for biocatalytic pyrrole synthesis. Beyond pyrrole synthesis, this work establishes ThrDHs as promising biocatalysts for nitrogen‐containing heterocycle synthesis and highlights dual‐function cosubstrates as an effective strategy for integrated cofactor recycling in chemoenzymatic cascades. Future engineering of ThrDHs, including recently reported consensus‐designed and computationally optimized variants [44, 45], together with ADH screening to further exploit the dual‐function cosubstrate concept, may expand the accessible α‐aminoketone intermediates and pyrrole substitution patterns.

## Author Contributions

**Valentina Jurkaš**: methodology, data curation, investigation, visualization, writing – original draft, supervision. **Fabian M. P. Kulier**: methodology, investigation. **Jorge González‐Rodríguez**: investigation, methodology, visualization, writing – original draft. **Carlotta Chiesa**: methodology, investigation. **Peter Both**: funding acquisition, project administration, resources, validation, writing – review and editing. **Peter Šiška**: validation, funding acquisition, resources, writing – review and editing, supervision. **Fabio Parmeggiani**: conceptualization, writing – review and editing, supervision, project administration. **Florian Rudroff**: conceptualization, supervision, project administration, writing – review and editing, data curation. **Margit Winkler**: conceptualization, funding acquisition, project administration, visualization, writing – review and editing, supervision.

## Conflicts of Interest

The authors declare no conflicts of Interest.

## Supporting information



The authors have cited additional references within the Supporting Information [46, 47, 48, 49, 50, 51].**Supporting File**: anie74918‐sup‐0001‐SI2_rev1_unlinked.docx.

## Data Availability

The data that supports the findings of this study are available in the Supporting Information of this article.
