# Supplementary material for: Dual‐Function Cosubstrates Enable Threonine Dehydrogenase‐Driven Chemoenzymatic Pyrrole Synthesis
Source: Angew Chem Int Ed Engl. 2026 Oct 1;65(46):e6159381. doi: 10.1002/anie.6159381 (PMC13749248; doi:10.1002/anie.6159381)
Supplement: Supplementary file 1 [file ANIE-65-e6159381-s001.docx]

Supporting Information

Dual-Function Cosubstrates Enable Threonine Dehydrogenase Driven Chemoenzymatic Pyrrole Synthesis

Valentina Jurkaš,^[a,b]^ Fabian M. Kulier,^[a,b]^ Jorge González-Rodríguez,^[a,c]^ Carlotta Chiesa,^[d]^ Peter Both,^[e]^ Peter Šiška,^[e]^ Fabio Parmeggiani,^[d]^ Florian Rudroff,^[a,c]^ Margit Winkler*^[a,f]^

[a]	Dr. Valentina Jurkaš, Fabian M. Kulier, Dr. Jorge González-Rodríguez, Prof. Dr Florian Rudroff, Prof. Dr. Margit Winkler

Austrian Centre of Industrial Biotechnology (ACIB), Krenngasse 37/2, 8010 Graz, Austria

[b]	Valentina Jurkaš,^[a,b]^ Fabian M. Kulier,^[a,b]^ Prof. Dr. Margit Winkler

Graz University of Technology, Institute of Molecular Biotechnology, Petersgasse 14, 8010 Graz, Austria

[c]	Dr. Jorge González-Rodríguez, Prof. Dr Florian Rudroff

TU Wien, Institute of Applied Synthetic Chemistry, Getreidemarkt 9/163-OC, 1060 Wien, Austria

[d]	Carlotta Chiesa, Prof. Dr. Fabio Parmeggiani

Politecnico di Milano, Dipartimento di Chimica, Materiali ed Ingegneria Chimica “Giulio Natta”, Piazza Leonardo Da Vinci 32, 20133 Milan, Italy

[e]	Dr. Peter Both, Peter Šiška Axxence Slovakia s.r.o., Mickiewiczova 9, 81107 Bratislava, Slovakia

[f]	Prof. Dr. Margit Winkler

Zurich University of Applied Sciences, Institute of Chemistry and Biotechnology, Einsiedlerstrasse 31, 8820 Wädenswil, Switzerland

*	Email: margit.winkler@zhaw.ch

# 1 Table of Contents

1	Table of Contents	1

2	Supplementary Data	3

2.1	SDS-PAGE of ThrDH purifications	3

2.1.1	SDS-PAGE of ThrDH purifications	3

2.1.2	Coexpression of *Ec*ThrDHs and ADHs	3

2.2	Spectrophotometric activity assays with purified ThrDHs	4

2.2.1	Specific activity of purified ThrDHs	4

2.2.2	Kinetic parameters of purified ThrDHs	4

2.3	Biotransformations	5

2.3.1	**2a** formation using purified ThrDHs	5

2.3.2	**2a** formation with ThrDH panel used as whole-cell catalyst	6

2.3.3	Dependence of **2a** formation on **1a** loading	6

2.3.4	Dependence of **2a** formation on NAD^+^ concentration	7

2.3.5	**2a** formation with different l-Thr loading	7

2.3.6	**2a** formation using a second whole cell catalyst overexpressing an ADH for NAD^+^ regeneration	8

2.3.7	**2a** formation using lysates of *Ec*ThrDH and ADH for NAD^+^ regeneration	8

2.3.8	**2a** formation using whole cells coexpressing *Ec*ThrDH and ADHs	9

2.3.9	Time course of 2a formation using whole cells coexpressing *Ec*ThrDH and ADHs	9

2.3.10	Dual-substrate scope	10

2.3.11	Oxidation of 1-aminopropan-2-ol	12

3	Experimental information	13

3.1	General, kits, instruments and chemicals	13

3.2	Enzymes	13

3.3	Cloning	14

3.4	Cultivation and enzyme expression in *E. coli*	16

3.4.1	Cultivation and recombinant protein expression.	16

3.4.2	Protein purification	17

3.4.3	Preparation of whole cells for use in biotransformations	18

3.4.4	Preparation of cell lysates for use in biotransformations	18

3.4.5	SDS-PAGE	18

3.5	Spectrophotometric activity assays	18

3.6	General procedure for biotransformation	19

3.6.1	Chemical formation of pyrrole **2a** in buffer	19

3.6.2	Biotransformation with purified enzymes	19

3.6.3	Biotransformation with whole-cells	19

3.6.4	Biotransformation with lysates	19

3.6.5	100 mL scale biotransformation for synthesis of pyrrole **2a**	19

3.7	Synthesis of pyrrole reference compounds	20

4	Analytics and Chromatographic Data	20

4.1	HPLC-MS	20

4.2	GC-FID	21

4.3	NMR	21

4.4	Representative chromatograms	28

4.5	Calibration curves	38

4.6	DNA sequences	39

4.6.1	*Cn*ThrDH	39

4.6.2	*Bs*ThrDH	39

4.6.3	*Ec*ThrDH	39

4.6.4	*Lok*ThrDH	39

4.6.5	ADH-A	39

4.6.6	ADH-hT	40

5	References	40

# 2 Supplementary Data

## 2.1 SDS-PAGE of ThrDH purifications

### 2.1.1 SDS-PAGE of ThrDH purifications

**Figure S1**. SDS-PAGE of the purification of ThrDHs using Ni-sepharose. *Cn*ThrDH, 35.7 kDa; *Bs*ThrDH, 36.5 kDa; *Ec*ThrDH, 38.4 kDa; *Lok*ThrDH, 38. 6 kDa. NuPage 4-12% Bis-Tris Gel; 175V, 75 min. **M**, Page Ruler, Thermo Scientific, #26616; **L**, lysate; **F**, flowthrough; **W1**, wash 1 (binding buffer); **W2**, wash 2 (washing buffer); **E**, elution; **D**, purified enzyme after desalting (7.5 µg). Brightness and contrast of the image were adjusted uniformly across the entire image to improve band visibility.

### 2.1.2 Coexpression of *Ec*ThrDHs and ADHs

**Figure S2.** Coexpression of *Ec*ThrDH and ADHs from the pETDuet-1 backbone. Expression in *E. coli* BL21(DE3), 0.5 mM IPTG, 20 ºC, overnight. Bolt 4-13% Bis-Tris Gel; 200 V, 45 min. *Ec*ThrDH, 38.4 kDa; ADH-A, 35.1 kDa; ADH-hT 36.3 kDa. S: Soluble fraction, P: Pellet, EVC, empty vector control, M: Page Ruler, Thermo Scientific, #26616. Brightness and contrast of the image were adjusted uniformly across the entire image to improve band visibility.

## 2.2 Spectrophotometric activity assays with purified ThrDHs

### 2.2.1 Specific activity of purified ThrDHs

specific activity [U mg-1]

|  | specific activity [U/mg] |
| --- | --- |
| CnThrDH | 19.3025 |
| BsThrDH | 20.211 |

specific activity [U mg-1]

|  | specific activity [U/mg] |
| --- | --- |
| EcThrDH | 2.10023 |
| LokThrDH | 1.24897 |

**Figure S3.** Specific activity of purified ThrDHs for the oxidation of l-Thr (10 mM) in the presence of NAD^+^ (0.5 mM), measured photometrically as increase in NADH absorbance at 340 nm in HEPES (100 mM, pH 8) at room temperature. Average and standard deviation of technical triplicates, background activity of control reactions without substrate subtracted.

### 2.2.2 Kinetic parameters of purified ThrDHs

**Table S1.** Overview of best buffers and kinetic parameters of ThrDHs toward l-Thr in KPi 100 mM, pH 7.5

|  | Enzyme | Best stability | Best reaction buffer | Activation by Mn^2+^ | *k*_cat_ [s^-1^] | *K*_M_ [mM] | *k*_cat_/*K*_M_ [s^-1^ mM^-1^] |
| --- | --- | --- | --- | --- | --- | --- | --- |
| SDR | *Cn*ThrDH | KPi (pH 7-9) | Tris-HCl (pH 9)  glycine/NaOH (pH 9) | +/- | 22.5 ± 1.0 | 5.4 ± 0.1 | 4.2 ± 0.1 |
|  | *Bs*ThrDH | KPi (pH 8) | KPi (pH 9) | - | 33.7 ± 0.8 | 6.1 ± 0.3 | 5.6 ± 0.1 |
| MDR | *Ec*ThrDH | KPi (pH 7-8) | Tris-HCl (pH 9)  glycine/NaOH (pH 11) | - | 1.5 ± 0.1 | 3.5 ± 0.2 | 0.4 ± 0.1 |
|  | *Lok*ThrDH | Glycine/NaOH (pH 9) | Tris-HCl  HEPES (pH 8) | + | 0.9 ± 0.1 | 7.5 ± 1.8 | 0.1 ± 0.0 |

SDR, short-chain dehydrogenase/reductase; MDR, medium-chain dehydrogenase/reductase

## 2.3 Biotransformations

**Scheme S1**. Concept of the ThrDH-driven chemoenzymatic pyrrole synthesis. ThrDH converts l-Thr into aminoacetone, which is intercepted by *β*-dicarbonyl compounds **1** in a Knorr pyrrole synthesis (KPS) to yield substituted pyrroles **2**. In the absence of efficient trapping by *β*-dicarbonyl compounds, aminoacetone readily undergoes oxidative dimerization to form 2,5-dimethylpyrazine (2,5-DMP)^[1,2]^ as a competing side product.

### 2.3.1 2a formation using purified ThrDHs

**Figure S4.** Biotransformation of l-Thr using purified ThrDHs. ThrDH (1 mg mL^-1^), l-Thr (10 mM), NAD^+^ (10 mM), **1a** (3% v/v) in in KPi (100 mM, pH 7.5) or Tris-HCl (100 mM + MnCl_2_ 1 mM; for *Lok*ThrDH), total reaction volume 0.5 mL. Incubated 18 h at 37 °C in 2 mL Eppendorf tubes with 120 rpm horizontal shaking.

### 2.3.2 2a formation with ThrDH panel used as whole-cell catalyst

**Figure S5.** Whole-cell catalyzed formation of **2a**. ThrDH (whole *E. coli* BL21(DE3), OD_600_ = 10), ʟ-Thr (10 or 50 mM), **1a** (3% v/v), NAD^+^ (1 mM), in KPi (100 mM, pH 7.5) or Tris-HCl (100 mM + MnCl_2_ 1 mM; for LokThrDH), total reaction volume 0.5 mL. Incubated 18 h at 37 °C in 2 mL microcentrifuge tubes positioned horizontally at 130 rpm. Average and standard deviation of technical triplicates, workup with 2 volumes of MeOH, samples analyzed on HPLC-MS.

### 2.3.3 Dependence of 2a formation on 1a loading

**Figure S6.** Whole-cell catalyzed formation of **2a** using different **1a** and cell loading. *Ec*ThrDH (resting *E. coli* BL21(DE3), OD_600_ as indicated), ʟ-Thr (50 mM), **1a** (1-5% v/v), NAD^+^ (1 mM) in KPi (100 mM, pH 7.5), total reaction volume 0.5 mL. Incubated 18 h at 37 °C in 2 mL microcentrifuge tubes positioned horizontally at 130 rpm. Average and standard deviation of technical triplicates, workup with 2 volumes of MeOH, samples analyzed on HPLC-MS.

### 2.3.4 Dependence of 2a formation on NAD^+^ concentration

**Figure S7.** Effect of external NAD^+^ supply on **2a** formation. *Ec*ThrDH (resting *E. coli* BL21(DE3), OD_600_ = 10), l-Thr (50 mM), **1a** (2% v/v), NAD^+^ (0-5 mM) in KPi (100 mM, pH 7.5), total reaction volume 0.5 mL, incubated 18 h at 37 °C in 2 mL microcentrifuge tubes positioned horizontally at 130 rpm. Average and standard deviation of technical triplicates, workup with 2 volumes of MeOH, samples analyzed on HPLC-MS.

### 2.3.5 2a formation with different l-Thr loading

**Figure S8.** Substrate concentration dependence of whole-cell catalyzed formation of **2a**. *Ec*ThrDH (resting *E. coli* BL21(DE3), OD_600_ = 10 or 20), l-Thr (50-300 mM), **1a** (2 or 4% v/v), NAD^+^ (1 mM) in KPi (100 mM, pH 7.5), total reaction volume 0.5 mL. Incubated 18 h at 37 °C in 2 mL microcentrifuge tubes positioned horizontally at 130 rpm. Average and standard deviation of technical triplicates, workup with 2 volumes of MeOH, samples analyzed on HPLC-MS. <2 mM 2,5-DMP detected per sample (not shown).

### 2.3.6 2a formation using a second whole cell catalyst overexpressing an ADH for NAD^+^ regeneration

**Figure S9.** Whole-cell catalyzed formation of **2a** and methyl 3-hydroxybutanoate (**3a**) using a second whole cell species overexpressing an enzyme for NAD^+^ regeneration. *Ec*ThrDH, ADH-A, ADH-hT or EVC -empty vector control- (resting *E. coli* BL21(DE3), OD_600_ = 10), l-Thr (50 mM), **1a** (2% v/v = 183 mM), NAD^+^ (1 mM) in KPi (100 mM, pH 7.5), total reaction volume 0.5 mL. Incubated 18 h at 37 °C at 130 rpm in 2 mL microcentrifuge tubes positioned horizontally. Average and standard deviation of technical triplicates, workup with 2 volumes of MeOH, samples analyzed on HPLC-MS.

### 2.3.7 2a formation using lysates of *Ec*ThrDH and ADH for NAD^+^ regeneration

**Figure S10.** Lysate-catalyzed formation of **2a** and methyl 3-hydroxybutanoate (**3a**) with ADHs for NAD⁺ recycling. Reaction conditions: *Ec*ThrDH, ADH-A, ADH-hT or EVC (lysate from *E. coli* BL21(DE3); each corresponding to OD_600_ = 10), NAD^+^ (1 mM) in KPi (100 mM, pH 7.5), total reaction volume 0.5 mL. Incubated 18 h at 37 °C at 130 rpm in 2 mL microcentrifuge tubes positioned horizontally. Average and standard deviation of technical triplicates, workup with 2 volumes of MeOH, samples analyzed on HPLC-MS. **A**: l-Thr (50 mM), **1a** (2% v/v); **B**: *Ec*ThrDH and ADH-A; l-Thr (300 mM), **1a** (2-15% v/v).

### 2.3.8 2a formation using whole cells coexpressing *Ec*ThrDH and ADHs

**Figure S11.** **2a** formation with whole cells coexpressing *Ec*ThrDH and ADHs. Whole *E. coli* BL21(DE3) corresponding to OD_600_ = 20, NAD^+^ (1 mM) in KPi (100 mM, pH 7.5), total reaction volume 0.5 mL. Incubated 18 h at 37 °C in 2 mL microcentrifuge tubes positioned horizontally in a shaker at 130 rpm. Average and standard deviation of technical triplicates, workup with 2 volumes of MeOH, samples analyzed on HPLC-MS. **A**: resting cells, ʟ-Thr (150 mM), **1a** (4% v/v); **B**: resting vs. freeze-thawed cells, coexpression from pETDuet_*Ec*ThrDH_ADH-A, ʟ-Thr (150 mM), **1a** (4% v/v); **C**: freeze-thawed cells, coexpression from pETDuet_*Ec*ThrDH_ADH-A, ʟ-Thr (300 mM), **1a** (4-10% v/v).

### 2.3.9 Time course of 2a formation using whole cells coexpressing *Ec*ThrDH and ADHs

**Figure S12.** Time course of **2a** formation with whole cells coexpressing *Ec*ThrDH and ADHs. Frozen & thawed whole *E. coli* BL21(DE3) pETDuet_*Ec*ThrDH_ADH-A (corresponding to OD_600_ = 20), ʟ-Thr (300 mM), **1a** (8% v/v), NAD^+^ (1 mM) in KPi (100 mM, pH 7.5), total reaction volume 0.5 mL. Incubated 24 h at 37 °C in 2 mL microcentrifuge tubes positioned horizontally in a shaker at 130 rpm. Individual samples were prepared for each time-point. Average and standard deviation of technical triplicates, workup with 2 volumes of MeOH, samples analyzed on HPLC-MS.

### 2.3.10 Dual-substrate scope

**Figure S13.** Structures of dicarbonyl cosubstrates used in this work.

**Figure S14.** Structures of pyrroles that were synthesized in this work.

 **Figure S15.** Comparison of pyrrole formation with different dicarbonyl cosubstrates using either aminoacetone (50 mM) or *Ec*ThrDH [*E. coli* BL21(DE3) resting cells, OD_600_ = 10] with l-Thr (50 mM) and NAD^+^ (1 mM). 2% v/v **1a-g**, or 100 mM **1h-i** in KPi (100 mM, pH 7.5), total reaction volume 0.5 mL. Incubated 18 h at 37 °C in 2 mL microcentrifuge tubes positioned horizontally at 130 rpm. Workup with 2 volumes of MeOH, samples analyzed on HPLC-MS.

**Figure S16**. Pyrrole formation with different dicarbonyl cosubstrates using optimized conditions for **2a** formation with (**a**) resting cells expressing only *Ec*ThrDH or (**c**) the coexpression strain. **a:** resting cells overexpressing *Ec*ThrDH from pK470, 4% v/v cosubstrate; **b:** resting cells overexpressing *Ec*ThrDH and ADH-A from pETDuet, 4% v/v cosubstrate **c:** frozen & thawed *E.coli* BL21(DE3) overexpressing *Ec*ThrDH and ADH-A from pETDuet, 8% v/v cosubstrate. Reaction conditions: whole *E. coli* BL21(DE3) cells (OD_600_ = 20), l-Thr (300 mM), cosubstrate **1a-g**, NAD^+^ (1 mM) in KPi (100 mM, pH 7.5), total reaction volume 0.5 mL. Incubated 18 h at 37 °C in 2 mL microcentrifuge tubes positioned horizontally at 130 rpm. Workup with 2 volumes of MeOH, samples analyzed on HPLC-MS.

HPLC peak area

|  | pyrrole | 2,5-DMP |  |
| --- | --- | --- | --- |
| 2a a | 0.984805M |  | 1 |
| 2a b | 2.9925M | 0.178469M |  |
| 2h a | 0.0458075M | 0.102569M |  |
| 2h b | 0.154296M | 0.097394M |  |
| 2i a | 0.071082M | 0.060955M |  |
| 2i b | 0.172684M | 0.0552575M |  |

**Figure S17**. Pyrrole formation with dicarbonyl cosubstrates **1h** and **1i** (100 mM) as compared to **1a** (4%) using resting cells overexpressing (**a**) *Ec*ThrDH from pK470 or (**b**) *Ec*ThrDH and ADH-A from pETDuet, Reaction conditions: whole *E. coli* BL21(DE3) cells (OD_600_ = 20), l-Thr (300 mM), NAD^+^ (1 mM) in KPi (100 mM, pH 7.5), total reaction volume 0.5 mL. Incubated 18 h at 37 °C in 2 mL microcentrifuge tubes positioned horizontally at 130 rpm. Workup with 2 volumes of MeOH, samples analyzed on HPLC-MS. **2h** and **2i** could not be quantified due to the lack of reference material. Peak areas for the reaction towards **2a** correspond to samples a and b depicted in **Figure S16**.

### 2.3.11 Oxidation of 1-aminopropan-2-ol

| **A** |  |
| --- | --- |
| **B** |  |

**Figure S18.** Oxidation of 1-aminopropan-2-ol. (**A**) 1-aminopropan-2-ol is oxidized to aminoacetone that can participate in the KPS. (**B**) Formation of pyrrole **2a** using oxidation of 1-aminopropan-2-ol as the source of aminoacetone under reaction conditions corresponding to the optimized conditions for **2a** formation. Reaction conditions: 1-aminopropan-2-ol (100 mM), **1a** (4% v/v), ADH-A or EVC (lysate from *E. coli* BL21(DE3); each corresponding to OD_600_ = 10), NAD^+^ (1 mM) in KPi (100 mM, pH 7.5), total reaction volume 0.5 mL. Incubated 16 h at 37 °C at 130 rpm in 2 mL microcentrifuge tubes positioned horizontally. Average and standard deviation of technical triplicate or a single measurement, workup with 2 volumes of MeOH, samples analyzed on HPLC-MS.

# 3 Experimental information

## 3.1 General, kits, instruments and chemicals

Optical densities of *E. coli* cell cultures were determined on an Implen OD_600_ DiluPhotometer. DNA and enzyme concentrations were determined on a NanoDrop. SDS page was performed with NuPAGE™ Bis-Tris Mini Protein Gels. General reagents, substrates and solvents were purchased and used as supplied from Sigma-Aldrich (Merck KGaA), Thermo Fisher Scientific, Fluka, Lancaster, Roth and BLDPharm. Unless stated otherwise, reagents and organic solvents were obtained from commercial suppliers in reagent grade quality and used without further purification. Commercially available substrates, products and coproducts are listed in **Table S2**. Synthesis of other reference compounds is described in section 3.7.

**Table S2.** List of analyzed commercially available substrates, products and coproducts.

| **#** | **chemical** | **CAS number** | **MW** | **purity** | **supplier** | **product ID** |
| --- | --- | --- | --- | --- | --- | --- |
|  |  |  | **[g mol^-1^]** | **[%]** |  |  |
| **l-Thr** | l-threonine | 72-19-5 | 119.12 | 98 | Sigma Aldrich | T8625 |
|  | aminoacetone HCl | 7737-17-9 | 109.55 | 95 | BDLpharm | BD113957 |
|  | 1-aminopropan-2-ol | 78-96-6 | 75.11 | 93 | Sigma Aldrich | 110248 |
| **1a** | methyl acetoacetate | 105-45-3 | 116.12 | 98.5 | Sigma Aldrich | 537365 |
| **3a** | methyl 3-hydroxybutanoate | 1487-49-6 | 118.13 | 97 | BLDPharm | BD766047 |
| **1b** | ethyl acetoacetate | 141-97-9 | 130.14 | 99 | SAFC | W241512 |
| **1c** | *tert*-butyl acetoacetate | 1694-31-1 | 158.20 | 97 | BLDPharm | BD152986 |
| **1d** | benzyl acetoacetate | 5396-89-4 | 192.21 | 98 | BLDPharm | BD116106 |
| **1e** | allyl acetoacetate | 1118-84-9 | 142.15 | 95 | BLDPharm | BD113063 |
| **1f** | methyl 3-oxovalerate | 30414-53-0 | 130.14 | 98 | BLDPharm | BD2919 |
| **1g** | acetyl acetone | 123-54-6 | 100.12 | 99 | Sigma Aldrich | P7754 |
| **2g** | 3-acetyl-2,4-dimethylpyrrole | 2386-25-6 | 137.18 | 98 | BLDPharm | BD5312 |
| **1h** | cyclohexane-1,3-dione | 504-02-9 | 112.13 | 97 | BLDPharm | BD33836 |
| **1i** | dihydro-2H-pyran-2,4(3H)-dione | 33532-46-6 | 114.10 | 95 | BLDPharm | BD295394 |

## 3.2 Enzymes

The enzymes used in this study are listed in Table S3.

Table S3. Enzymes used in this work.

| type | enzyme | source organism | accession number |
| --- | --- | --- | --- |
| SDR | *Cn*ThrDH^[1,3]^ | *Cupriavidus necator* | BAJ61594.1 |
|  | *Bs*ThrDH^[4,5]^ | *Bacillus subtilis* | CUB51851.1 |
| MDR | *Ec*ThrDH^[6–8]^ | *Escherichia coli* | P07913.1 |
|  | *Lok*ThrDH^[9]^ | *Loktanella* sp. | WP_129681835.1 |
|  | ADH-A^[10–12]^ | *Rhodococcus ruber* DSM 44541 | WP_043801412.1 |
|  | ADH-hT^[13]^ | *Geobacillus stearothermophilus* | P42328.1 |

SDR, short-chain dehydrogenase; MDR, medium-chain dehydrogenase.

## 3.3 Cloning

DNA sequences for genes encoding enzymes used in this work are listed in section 4.6.

ThrDHs flanked by pK470/pM470 vector sequences were purchased as gene fragments from Twist Bioscience and the *Ec*ThrDH was amplified from *E. coli* TOP 10 F’ genome. For subcloning of genes into pETDuet-1, the genes were amplified with 40bp-flanks corresponding to regions flanking MCS1 or MCS2 of the pETDuet-1 vector. Linear K470 and pETDuet-1 backbones were generated by PCR with corresponding flanks. All the PCR reactions are listed in **Table S4** and all the primer sequences in Table S5. PCR amplification was done using Q5^®^ High-Fidelity DNA Polymerase (New England Biolabs), according to the manufacturer’s instructions (**Table S6** and **Table S7**). The PCR products were purified from agarose gel using Wizard^®^ SV Gel and PCR Clean-Up System (Promega).

For assembly, the backbone (100 ng) and insert (100 ng) were added to Gibson Mix (15 µL), final reaction volume 20 µL, according to Table S8. The reaction was incubated for 1h at 50°C, desalted for 30 min using the MF-Milipore 0.025 µM MCE membrane. Electrocompetent *E. coli* TOP 10 F’ was transformed with desalted DNA and grown overnight at 37°C on LB agar plates supplemented with the corresponding antibiotic.

Single colonies were picked and grown overnight in LB medium (10 mL) supplemented with the corresponding antibiotic, the plasmid isolated using Wizard^®^ Plus SV Minipreps DNA Purification System (Promega) and sequenced (Microsynth Austria) using primers pET-up, DSC37 and T7term. All the plasmids used and prepared in this work are listed in **Table S8**.

**Table S4.** List of PCR reactions for amplification of inserts and backbones for cloning into pETDuet-1 vector backbone.

| **PCR** | **template** | **primers** | | **PCR product** | |
| --- | --- | --- | --- | --- | --- |
|  |  | **forward** | **reverse** | **name** | **size [bp]** |
| **1** | *E. coli* TOP 10 F’ | #96 | #97 | *Ec*ThrDH_genomic | 1070 |
| **2** | *Ec*ThrDH_genomic | #99 | #100 | *Ec*ThrDH-His6 |  |
| **3** | pK470_ADH-A | #68 | #67 | pK470_linear | 4000 |
| **4** | pETDuet-1 (pWIM58) | #63 | #27 | BB_pETDuet_MCS1 | 5322 |
| **5** | pETDuet-1 (pWIM58) | #117 | #118 | BB_pETDuet_MCS2 | 5322 |
| **6** | pK470_*Ec*ThrDH (pWIM78) | #110 | #112 | *Ec*ThrDH(MCS1) | 1150 |
| **7** | pK470_ADH-A (pWIM2) | #64 | #114 | ADH-A(MCS1) | 1129 |
| **8** | pK470_ADH-hT (pWIM4) | #64 | #116 | ADH-hT(MCS1) | 1111 |
| **9** | pK470_*Ec*ThrDH (pWIM78) | #121 | #123 | *Ec*ThrDH(MCS2) | 1145 |
| **10** | pK470_ADH-A (pWIM2) | #124 | #125 | ADH-A(MCS2) | 1124 |
| **11** | pK470_ADH-hT (pWIM4) | #126 | #127 | ADH-hT(MCS2) | 1106 |
| **12** | pETDuet-1_*Ec*ThrDH(MCS1) | #117 | #118 | pETDuet-1_*Ec*ThrDH_MCS2 | 6260 |
| **13** | pETDuet-1_ADH-A(MCS1) | #117 | #118 | pETDuet-1_ADH-A_MCS2 | 6240 |
| **14** | pETDuet-1_ADH-hT(MCS1) | #117 | #118 | pETDuet-1_ADHhT _MCS2 | 6222 |

Table S5. Primers used in this work

| **Primer Name** | **DNA sequence 5´-3´** | **Purpose** |
| --- | --- | --- |
| #25 petDuet_MCS1_fwd | CTTTAAGAAGGAGATATACC | colony PCR – amplification of insert from pETDuet-1 MCS1 |
| #26 pETDuet_MCS1_rev | GATGAGAACCCCCCATGGTATATCTCCTTCTTAAAG | colony PCR – amplification of insert from pETDuet-1 MCS1 |
| #27 pETDuet_GB_MC1_fwd | GTCGAACAGAAAGTAATCGTATTGTACACGGCCGCATAATC | amplification of pETDuet-1 backbone  with flanks for cloning into MCS1 |
| #63 pETDuet_MCS1_rev2 | GTATATCTCCTTCTTAAAGTTAAACAAAATTATTTCTAGAGG | amplification of pETDuet-1 backbone  with flanks for cloning into MCS1 |
| #64 pETDuet_MCS1_fwd2 | CCTCTAGAAATAATTTTGTTTAACTTTAAGAAGGAGATATAC | amplification of insert from the pK470 backbone for cloning into pETDuet-1 MCS1 |
| #67 pK470_rev | CATGGTGGTGGTGATGATGCATATGTATATCTCCTTCTTAAAG | amplification pK470 backbone |
| #68 pK470_fw | GCTTGGCTGTTTTGGCGGATGAGAGAAG | amplification pK470 backbone |
| #96 EcThrDH_fw | CGTTATCGCCTGAGGATGTGAG | amplification of EcThrDH from genome |
| #97 EcThrDH_rev | GGAATACCAGCCCTTGTTCGTG | amplification of EcThrDH from genome |
| #99 EcThrDH_fw3_His | ATGCATCATCACCACCACCATGGTTCCGGTATGAAAGCGTTATCCAAACTG | adding N-term His-tag |
| #100 EcThrDH_rev2 | GAAAATCTTCTCTCATCCGCCAAAACAGCCAAGCTCATTAATCCCAGCTCAGAATAAC | adding pK470 downstream flank |
| #112 EcThDH_MCS1_rev | GATTATGCGGCCGTGTACAATACGATTACTTTCTGTTCGACTTAAGCATCATTAATCCCAGCTCAGAATAAC | amplification of insert with flanks for cloning into pETDuet-1 MCS1 |
| #114 ADH-A_MCS1_rev | GATTATGCGGCCGTGTACAATACGATTACTTTCTGTTCGACTTAAGCATTACGGCACCACCACGCCG | amplification of insert with flanks for cloning into pETDuet-1 MCS1 |
| #116 ADH-hT_MCS1_rev | GATTATGCGGCCGTGTACAATACGATTACTTTCTGTTCGACTTAAGCATTATTTATCTTCCAGGGTCAGC | amplification of insert with flanks for cloning into pETDuet-1 MCS1 |
| #117 pETDuet_MCS2_rev | ATGTATATCTCCTTCTTATACTTAACTAATATACTAAGATGG | amplification of pETDuet-1 backbone with flanks for cloning into MCS2 |
| #118 pETDuet_MCS2_fw | TTAACCTAGGCTGCTGCCACCGCTGAGCAATAACTAGCATAACC | amplification of pETDuet-1 backbone with flanks for cloning into MCS2 |
| #123 EcThrDH_MCS2_rev | GGTTATGCTAGTTATTGCTCAGCGGTGGCAGCAGCCTAGGTTAATCATTAATCCCAGCTCAGAATAAC | amplification of insert with flanks for cloning into pETDuet-1 MCS2 |
| #124 ADH-A_MCS2_fw | CCATCTTAGTATATTAGTTAAGTATAAGAAGGAGATATACATATGAAAGCGGTGCAGTATACC | amplification of insert with flanks for cloning into pETDuet-1 MCS2 |
| #125 ADH-A_MCS2_rev | GGTTATGCTAGTTATTGCTCAGCGGTGGCAGCAGCCTAGGTTAATTACGGCACCACCACGCCG | amplification of insert with flanks for cloning into pETDuet-1 MCS2 |
| #126 ADH-hT_MCS2_fw | CCATCTTAGTATATTAGTTAAGTATAAGAAGGAGATATACATATGAAAGCGGCGGTGGTGGAAC | amplification of insert with flanks for cloning into pETDuet-1 MCS2 |
| #127 ADH-hT_MCS2_rev | GGTTATGCTAGTTATTGCTCAGCGGTGGCAGCAGCCTAGGTTAATTATTTATCTTCCAGGGTCAGC | amplification of insert with flanks for cloning into pETDuet-1 MCS2 |
| pET-up | ATGCGTCCGGCGTAG | sequencing |
| DSC #37 pETDuet1_fwd | CCCCATCTTAGTATATTAGTTAAGTATAAGAAGGAG | sequencing |
| T7term | TGCTAGTTATTGCTCAGCGG | sequencing |

**Table S6.** Components and concentrations for PCR reactions using Q5^®^ High Fidelity DNA polymerase.

| **Component** | **V [µL]** | **Final concentration** |
| --- | --- | --- |
| 5X Q5 Reaction Buffer | 10 | 1x |
| 10 mM dNTPs | 2.5 | 500 µM |
| 10 µM Forward Primer | 2 | 0.4 µM |
| 10 µM Reverse Primer | 2 | 0.4 µM |
| Template DNA | variable | 1-10 ng |
| Q5 High-Fidelity DNA polymerase | 0.5 | 0.02 U/µL |
| Nuclease free water | to 50 µL |  |

**Table S7.** Thermocycling conditions for PCR amplification.

| **PCR program** | | |  | |
| --- | --- | --- | --- | --- |
| **no. of cycles** | | **step** | **T [°C]** | **time** |
| 1x | | initial denaturation | 98 | 30 sec |
| 30 x | | denaturation | 98 | 15 sec |
|  | | annealing | 56 | 30 sec |
|  | | extension | 72 | 30-50 sec/kba |
| 1x | | final extension | 72 | 10 min |
|  |  | hold | 4 | ∞ |

**Table S8.** List of plasmids used in this work.

| **plasmid** | **source** | **PCR products used for Gibson Assembly** | | **confirmed clone** |
| --- | --- | --- | --- | --- |
|  |  | **insert** | **backbone** |  |
| pK470_ADH-A | in house | not applicable | | |
| pK470_ADHhT | in house | not applicable | | |
| pMS470_*Cn*ThrDH | in house | not applicable | | |
| pK470_*Bs*ThrDH | this work | synthetic gene | BB_pK470 | VJU-B-102-06 |
| pK470_*Ec*ThrDH | this work | synthetic gene | BB_pK470 | VJU-B-093-08 |
| pK470_*Lok*ThrDH | this work | synthetic gene | BB_pK470 | VJU-B-102-04 |
| pETDuet-1_*Ec*ThrDH(MCS1) | this work | *Ec*ThrDH(MCS1) | BB_pETDuet_MCS1 | VJU-C-057-GA3-2 |
| pETDuet-1_ADH-A(MCS1) | this work | ADH-A(MCS1) | BB_pETDuet_MCS1 | VJU-C-062-4 |
| pETDuet-1_ADH-hT(MCS1) | this work | ADH-hT(MCS1) | BB_pETDuet_MCS1 | VJU-C-062-3 |
| pETDuet-1_*Ec*ThrDH_ADH-A | this work | ADH-A(MCS2) | pETDuet-1_*Ec*ThrDH_MCS2 | VJU-C-065-GA-07-3 |
| pETDuet-1_ADH-A_*Ec*ThrDH | this work | *Ec*ThrDH(MCS2) | pETDuet-1_ADH-A_MCS2 | VJU-C-065-GA-10-02 |
| pETDuet-1_ADH-hT_*Ec*ThrDH | this work | *Ec*ThrDH(MCS2) | pETDuet-1_ADHhT _MCS2 | VJU-C-065-GA-12-01 |
| pETDuet-1_*Ec*ThrDH(MCS2) | this work | *Ec*ThrDH(MCS2) | BB_pETDuet_MCS2 | VJU-C-067-trafo03-1 |

## 3.4 Cultivation and enzyme expression in *E. coli*

The enzymes used in this study were expressed in *E. coli* BL21(DE3).

### 3.4.1 Cultivation and recombinant protein expression.

Electrocompetent *E. coli* BL21(DE3) were transformed with the verified plasmids and plated on LB plates with the appropriate antibiotic (Table S9). Single colonies or glycerol stocks were used for inoculation of sterile LB-medium (10 mL, supplemented with the appropriate antibiotic) and grown overnight at 37 °C with shaking. The overnight cultures were used for the inoculation of sterile LB medium (1% v/v) supplemented with the corresponding antibiotic and incubated at 37 °C at 120 rpm. At OD_600_ 0.6 - 0.8 expression was induced by adding 0.5 mM IPTG, the temperature was lowered to 20 ºC and cultures further incubated for 20 h at 120 rpm.

**Table S9.** Cultivation conditions and absorbance for the enzymes expressed *E. coli* BL21(DE3) in this work.

| **name** | **plasmid backbone** | **medium** | **antibiotic** | **c_final_**  **[μg mL^-1^]** | **inducer/**  **c_final_ [mM]** | **T_expression_ [°C]** | **MW [kDa]** | **Abs 0.1%**  **(1 g l^-1^)^a^** |
| --- | --- | --- | --- | --- | --- | --- | --- | --- |
| *Cn*ThrDH | pMS470 | LB | Amp | 100 | IPTG/  0.5 | 20 | 35.7 | 1.734 |
| *Bs*ThrDH | pK470 |  | Kan | 50 |  |  | 36.5 | 1.024 |
| *Ec*ThrDH | pK470 |  | Kan | 50 |  |  | 38.4 | 1.092 |
| *Lok*ThrDH | pK470 |  | Kan | 50 |  |  | 38.6 | 1.113 |
| ADH-A | pK470 |  | Kan | 50 |  |  | 35.1 |  |
| ADH-hT | pK470 |  | Kan | 50 |  |  | 36.3 |  |
| coexpression constructs | pETDuet-1 |  | Amp | 100 |  |  | - |  |

Amp: ampicillin (c_stock_ = 100 mg mL^-1^, in H_2_O); Kan: kanamycin (c_stock_ = 50 mg mL^-1^, in H_2_O); IPTG: isopropyl-*β*-d-thiogalactopyranoside (c_stock_= 1M, in H_2_O); LB: NaCl (5 g L^-1^), yeast extract (5 g L^-1^), tryptone (10 g L^-1^).^a^based on extinction coefficients of fully reduced ThrDHs calculated using Benchling.

### 3.4.2 Protein purification

**Harvesting**. To harvest the cells, cultures were centrifuged for 20 min at 3220 g and 4 °C, the cell pellet resuspended in NaPi (10 mM, pH 7.4) and then centrifuged again under the same conditions. The pellets were stored at -20 °C.

Buffers used for purification are listed in Table S10.

**Cell lysis.** Harvested cell pellets were resuspended in binding buffer (5 mL g_pellet_^-1^) and sonicated in an ice water bath (Branson Sonifier 250; timer 3 min, duty cycle 50%, output control 7, 3 x with a 1 min break in between). The cell suspension was centrifuged (45 min, 20 000 rpm, 4 °C), and the supernatant filtered (0.45 µm, then 0.22 µm syringe filter) and kept on ice.

**His-tag purification**. ThrDHs were purified by an immobilized metal ion affinity chromatography (Ni-sepharose 6 Fast Flow, GE Healthcare), with gravity flow. The purification was performed at RT using buffers kept on ice. The column was equilibrated with 5 CV binding buffer, the filtered lysate was loaded onto the column and the column was washed with 5 CV binding buffer. Then, the column was washed with 5 CV washing buffer. The protein was eluted with 6 mL elution buffer. The column was washed with 5 CV elution buffer, followed by 5 CV of binding buffer and 10 CV of 20% ethanol. The column was stored at 4 °C.

**Storage.** The volume of the elution fraction was reduced to 2.5 mL using a Vivaspin^®^ 6 mL Ultrafiltration Unit (Sartorius). Then, the buffer was changed to storage buffer using a Sephadex G-25 PD10 desalting column (GE Healthcare). Protein concentration was determined measuring A_280_ on the Nanodrop and corrected using the protein extinction coefficient calculated using Benchling (Table S9). The final enzyme solution was aliquoted and stored at -20 °C.

Table S10. Buffers used for protein purification

| **enzyme** | **binding buffer** | **washing buffer** | **elution buffer** | **storage buffer** |
| --- | --- | --- | --- | --- |
| **ThrDHs** | 20 mM HEPES,  150 mM NaCl,  10 mM imidazol,  pH 7.5 | 20 mM HEPES,  150 mM NaCl,  30 mM imidazol,  pH 7.5 | 20 mM HEPES,  150 mM NaCl,  300 mM imidazol,  pH 7.5 | 50 mM HEPES,  150 mM NaCl,  pH 7.5 |

### 3.4.3 Preparation of whole cells for use in biotransformations

The cells were harvested by centrifugation (20 min at 3220 g and 4 °C), the supernatant discarded and the cell pellets immediately resuspended in the reaction buffer for use as resting cells or frozen and stored at -20 ºC for later use as “frozen and thawed” cells.

### 3.4.4 Preparation of cell lysates for use in biotransformations

The cells were harvested by centrifugation (20 min at 3220 g and 4 °C), the supernatant discarded and the cell pellets resuspended in the reaction buffer to OD_600_ of 50. The cell suspension was sonicated in an ice water bath (Branson Sonifier 250; timer 3 min, duty cycle 50%, output control 7, 3 x with a 1 min break in between). The cell debris were removed by centrifugation (45 min, 20 000 rpm, 4 °C) and lysates stored at -20 °C.

### 3.4.5 SDS-PAGE

The culture aliquots corresponding to 10 OD_600_ units were harvested. Cell pellets were resuspended in 100 μL of room temperature BugBuster® Protein Extraction Reagent (Milipore). Cell suspension was incubated 20 min at 25 °C and 600 rpm and centrifuged at 15 000 rpm for 20 min at 4°C. The supernatants correspond to soluble fractions. Pellets were resuspended in urea (6 M, 100 μL), incubated 20 min at 60°C and 200 rpm and centrifuged at 15 000 rpm for 20 min at 4°C. The supernatant corresponded to the insoluble fraction.

Protein concentration of purified and desalted enzymes was determined measuring A_280_ on the Nanodrop. Protein samples (10 µL; purification fractions of unknown concentration or purified enzyme at 3 g L^-1^) were mixed with the LDS Sample Buffer (4x) and Sample Reducing Agent (10x) to the final volume of 20 µL and heated to 80 °C for 8 minutes. Prepared samples (5 µL) and a marker (PageRuler Prestained Protein Ladder, Thermo Scientific, #26616; 5 µL) were loaded onto the NuPage 4-12% Bis-Tris Gel (175 V, 75 min, MOPS buffer). Gels were stained for 30 min using the “Magic” Coomassie staining solution (Coomassie Blue G-250, 110 mg; ethanol, 50 g; phosphoric acid, 80 g; dH_2_O, 850 mL; *β*-cyclodextrin, 10 g) and destained in deionized water overnight.

## 3.5 Spectrophotometric activity assays

Cofactors and substrates were prepared in HEPES (100 mM, pH 8) unless stated otherwise. The purified ThrDH (10 μL) was mixed with HEPES (40 µL) and NAD^+^ (2 mM, 50 μL; final concentration 0.5 mM) in a 96-well plate. The reaction was started by the addition of **l**-Thr (20 mM, 100 μL; final concentration 10 mM), final reaction volume of 200 μL. After 30 sec of initial shaking, the increase in absorption of NAD(P)H at 340 nm was followed for 10 min at room temperature (28 °C). For activity assays in alternative buffer systems, 10 μL of purified ThrDH was mixed with 100 μL of the appropriate buffer (200 mM) to account for subsequent dilution. Cofactors and substrates (NAD⁺ and **l**-Thr) were prepared in water and added to yield the same final concentrations as above (0.5 mM NAD⁺, 10 mM **l**-Thr, 100 mM final buffer concentration). One unit of enzyme activity (U = μmol min^-1^) is defined as the amount of the enzyme that catalyzes the generation of one μmol of NADH per minute under the specified conditions of the assay method. Specific enzyme activity is defined as the enzyme activity units per milligram of total protein (U mg^-1^ protein). Average and standard deviation of specific activity were calculated based on technical triplicates and the background activity of control reactions without substrate was subtracted.

## 3.6 General procedure for biotransformation

### 3.6.1 Chemical formation of pyrrole 2a in buffer

Aminoacetone hydrochloride (111 mM) was dissolved in an aqueous NaOH solution (110 mM). 225 μL (final conc. 51 mM) were mixed with 250 μL of the appropriate buffer (200 mM) and **1a** (15 μL, final conc. 3% v/v), final volume 0.49 mL in 2 mL-Eppendorf tubes shaken in horizontal position for 18 h at 37 °C and 120 rpm.

### 3.6.2 Biotransformation with purified enzymes

Cofactors and substrates were prepared in water. Enzymes (1 mg mL^-1^) were incubated with **l**-Thr (10 mM), **1a** (15 μL, final conc. 3% v/v), final volume 0.5 mL in 2 mL-Eppendorf tubes shaken in horizontal position for 18 h at 37 °C and 120 rpm.

### 3.6.3 Biotransformation with whole-cells

Cofactors, substrates and cells stocks were prepared in potassium phosphate buffer (100 mM, pH 7.5) and the cosubstrates were added neat. Setup for a representative biotransformation is described in **Table S11**. The reactions of final volume 0.5 mL were incubated in 2 mL-Eppendorf tubes, shaken in horizontal position for 18 h at 37 °C and 120 rpm.

**Table S11.** Example of stock solutions for preparation of pyrrole-forming biotransformation. Final volume was 500 µL.

| component | c_stock_ | V_per reaction_ [µL] | c_final_ |
| --- | --- | --- | --- |
| l-Thr | 750 mM | 200 | 300 mM |
| **1a** | neat | 40 | 8% v/v |
| NAD^+^ | 20 mM | 25 | 1 mM |
| *E. coli* BL21(DE3) | OD_600_ = 50 | 200 | OD_600_ = 20 |
| buffer |  | 35 |  |

### 3.6.4 Biotransformation with lysates

Lysates were produced from cell suspension corresponding to OD_600_ 50 and used in the reactions analogously to whole cell stocks (described in section 3.6.3).

### 3.6.5 100 mL scale biotransformation for synthesis of pyrrole 2a

l-Thr (3.67 g, final c. 300 mM), NAD^+^ (140 mg; final c. 2 mM) and **1a** (8 mL; final c. 8% v/v) were dissolved in 82 mL of KPi (100 mM, pH 7.5) directly in the reaction vessel (a baffled 250 mL Erlenmeyer flask with a screw neck). NAD⁺ was used at 2 mM for the preparative reaction as a precaution to ensure sufficient cofactor availability throughout the reaction (efficient NAD⁺ recycling had already been demonstrated at 1 mM on analytical scale). Frozen and thawed cell pellet corresponding to 2000 OD_600_ units was resuspended in 10 mL of KPi and combined with other reaction components in a final volume of 100 mL (final OD_600_ = 20). The Erlenmeyer flasks were closed with screw caps and incubated at 37 °C and 120 rpm for 24 h.

The crude mixture was cooled to 4 °C, filtered through a Büchner funnel and the solid washed three times with distilled water and collected in a flask. The liquid phase was left at 4 °C overnight, then, the filtering process was repeated, and the two solid fractions were collected in the same flask, which was left drying for 24 h under high vacuum, yielding 4.215 g of the pure product. As the remaining aqueous phase still contained some product, it was subjected to the work-up and purification described for the standard synthesis, yielding 47 mg of the pure product. In total, 4.262 g of the product were isolated, accounting for 93% yield. The identity and purity of the product were confirmed through 1H-NMR and 13C-NMR.

## 3.7 Synthesis of pyrrole reference compounds

Aminoacetone hydrochloride (50 mM) was dissolved in a mixture of 17 mL of KPi buffer (100 mM pH 8) containing 2.5% *vol* acetonitrile and flushed with argon for 45 minutes. Then, the corresponding dicarbonyl **1** was added, the pH was adjusted to 10 employing a solution of NaOH 10 M and the mixture was left to stir at 80 °C. The pH was checked and adjusted back to 10 three times per hour during the first three hours. Then, it was left to stir overnight. The crude mixture was then extracted, evaporated to dryness and purified through reverse phase chromatography in acidic mode, employing a Büchi Pure C-850 FlashPrep equipped with a Waters Atlantis C-18 preparative column, using mixtures of water and acetonitrile, obtaining pure pyrroles **2a-d** and **2f**. Chemical synthesis of **2e** was unsuccessful, therefore the synthesis of **2e** was performed as 20-mL upscale of the chemoenzymatic cascade, analogous to section 3.6.5., and followed the same purification protocol obtaining 423 mg (39% isolated yield). ^1^H-NMR, ^13^C-NMR data of compounds **2a, c-e**,^[14]^ **2b,**^[15]^ and **2f**^[16]^ matched with the ones of the commercially available products and is available in the section 4.3.

**methyl 2,4-dimethyl-1*H*-pyrrole-3-carboxylate (2a)**: ^1^H-NMR (400 MHz, CDCl_3_) δ 8.75 (s, 1H), 6.37 (s, 1H), 3.83 (s, 3H), 2.50 (s, 3H), 2.27 (s, 3H). ^13^C-NMR (100 MHz, CDCl_3_) δ 167.21 (C), 136.35 (C), 121.40 (C), 114.57 (C), 110.32 (CH), 50.51 (CH_3_), 14.00 (CH_3_), 12.61 (CH_3_).

**ethyl 2,4-dimethyl-1*H*-pyrrole-3-carboxylate (2b)**: ^1^H-NMR (400 MHz, CDCl_3_) δ 8.48 (s, 1H), 6.36 (s, 1H), 4.29 (q, *J* = 7.1 Hz, 2H), 2.49 (s, 3H), 2.26 (s, 3H), 1.37 (t, *J* = 7.1 Hz, 3H). ^13^C-NMR (100 MHz, CDCl_3_) δ 166.60 (C), 136.09 (C), 121.47 (C), 114.37 (C), 110.61 (CH), 59.32 (CH_2_), 14.51 (CH_3_), 14.04 (CH_3_), 12.64 (CH_3_).

***tert*-butyl 2,4-dimethyl-1*H*-pyrrole-3-carboxylate (2c)**: ^1^H-NMR (400 MHz, CDCl_3_) δ 8.31 (s, 1H), 6.35 (s, 1H), 2.47 (s, 3H), 2.24 (s, 3H), 1.58 (s, 9H). ^13^C-NMR (100 MHz, CDCl_3_) δ 165.90 (C), 135.47 (C), 121.29 (C), 114.15 (C), 112.03 (CH), 79.25 (C), 28.63 (3 x CH_3_), 14.12 (CH_3_), 12.78 (CH_3_).

**benzyl 2,4-dimethyl-1H-pyrrole-3-carboxylate (2d)**: ^1^H-NMR (400 MHz, CDCl_3_) δ 8.11 (s, 1H), 7.47–7.44 (m, 2H), 7.41–7.36 (m, 2H), ), 7.35–7.32 (m, 1H), 6.37 (s, 1H), 5.32 (s, 2H), 2.50 (s, 3H), 2.27 (s, 3H). ^13^C-NMR (100 MHz, CDCl_3_) δ 166.04 (C), 137.06 (C), 136.33 (C), 128.47 (2 x CH), 127.95 (2 x CH), 127.78 (CH), 121.76 (C), 114.34 (C), 110.48 (CH), 65.09 (CH_2_), 14.27 (CH_3_), 12.79 (CH_3_).

**allyl 2,4-dimethyl-1*H*-pyrrole-3-carboxylate (2e)**: ^1^H-NMR (400 MHz, CDCl_3_) δ 8.66 (s, 1H), 6.37 (s, 1H), 6.05 (ddt, *J* = 17.3, 10.7, 5.4 Hz, 1H), 5.40 (dq, *J* = 17.2, 1.6 Hz, 1H), 5.26 (dq, *J* = 10.5, 1.4 Hz, 1H), 4.77 (dt, *J* = 5.4, 1.6 Hz, 2H), 2.51 (s, 3H), 2.28 (d, *J* = 1.2 Hz, 3H). ^13^C NMR (100 MHz, CDCl_3_) δ 166.30 (C), 136.51 (C), 133.12 (CH), 121.53 (C), 117.25 (CH_2_), 114.58 (C), 110.25 (CH), 64.03 (CH_2_), 14.15 (CH_3_), 12.75 (CH_3_).

**methyl 2-ethyl-4-methyl-1*H*-pyrrole-3-carboxylate (2f)**: ^1^H-NMR (400 MHz, CDCl_3_) δ 7.94 (s, 1H), 6.30 (s, 1H), 3.73 (s, 3H), 2.87 (q, *J* = 7.6 Hz, 2H), 2.16 (d, *J* = 1.0 Hz, 3H), 1.16 (t, *J* = 7.6 Hz, 3H). ^13^C-NMR (100 MHz, CDCl_3_) δ 166.57 (C), 141.77 (C), 121.68 (C), 114.26 (C), 109.82 (CH), 50.41 (CH_2_), 21.25 (CH_3_), 13.36 (CH_3_), 12.59 (CH_3_).

# 4 Analytics and Chromatographic Data

## 4.1 HPLC-MS

For HPLC-MS analysis, an LCMS-2020 system from Shimadzu equipped with a DGU-20A3/5 degasser, LC-30AD Nexera pump, SIL-30AC Nexera autosampler, CTO-20AC thermostated column compartment, CBM-20A communication device and SPD-M20A detector was used. The analysis was carried out with a Macherey-Nagel EC 150/3 NUCLEODUR C18 Gravity 3 µm column equipped with a CC 8/3 NUCLEODUR C18 gravity 3 µm guard column

**HPLC method for detection of pyrroles.** Reaction mixture was diluted with 2 volumes of MeOH, vortexed and centrifuged for 40 min at 4° C and maximum speed and the supernatant was collected.. The mobile phases were H_2_O with formic acid (0.1% v/v) and ACN at a flowrate of 1.0 mL min^−1^. The following program was used for elution: 10% ACN for 0-0.7 min, gradient to 90% ACN for 0.7-3.1 min, 90% ACN for 3.1-4.8 min and 10% ACN from 4.81-6.0 min. 2,5-dimethylpyrazine was detected at 280 nm at 2.29 min (*m/z* 109 [M+1], 150 [M+42]). Pyrroles were detected at 234 nm: **2a**, 3.10 min (*m/z* 154 [M+1], 195 [M+42]); **2b**, 3.31 min (m/z 168 [M+1], 209 [M+42]); **2c**, 3.68 min (m/z 196 [M+1], 237 [M+42]); **2d**, 3.68 min (m/z 230 [M+1], 271 [M+42]); **2e**, 3.42 min (m/z 180 [M+1], 221 [M+42]); **2f** 3.30 min (m/z 168 [M+1], 209 [M+42]); **2g** 2.74 min (m/z 138 [M+1], 179 [M+42]); **2h** 2.60 min (m/z 150 [M+1], 191 [M+42]); **2i** 2.45 (m/z 152 [M+1], 193 [M+42]). All masses were detected in positive ionization mode.

**HPLC method for detection of benzoylated amines.** To the reaction mixture (40 μL) Na_2_CO_3_/NaHCO_3_ (0.2 M, pH 10.5, 100 µL), and benzoyl chloride (BzCl, 200 mM in ACN, 60 μL) were added. The mixture was vortexed for 30 sec and then quenched by the addition of HCl (1 M, 40 μL) and a solution of H_2_O:ACN (1:1; 60 μL). After centrifugation (40 min, 4°C, 15 000 rpm) the mixture was analysed by HPLC-MS. The mobile phases were H_2_O with ammonium acetate (5 mM) and ACN at a flow‐rate of 1.0 mL min^−1^. The following program was used for elution: 10% ACN for 0-0.7 min, gradient to 90% ACN for 0.7-3.1 min, 90% ACN for 3.1-4.8 min and 10% ACN from 4.8-6.0 min. Benzoylated compounds were detected at 228 nm. Retention times of benzoylated compounds: 1-aminopropan-2-ol, 2.47 min (m/z 180 [M+1]); aminoacetone 2.6 min (m/z 178 [M+1]).

## 4.2 GC-FID

The reaction mixtures (500 µL) were extracted with ethyl acetate (500 µL). The organic phase was dried over anhydrous sodium sulphate and compounds were quantified on GC-FID as their corresponding trimethylsilyl esters. For derivatization the organic phase (100 μL) was mixed with pyridine (60 μL) and BSTFA (60 μL), and incubated at room temperature for 20 min. Samples were measured on a GC 2010 Plus (Shimadzu) equipped with a ZB-5 column (30 m x 0.32 mm x 0.25 μm), with the following temperature program: 50 °C for 4 min, 40 °C min^-1^ to 300°C, hold 2 min, split ratio 10:1, injection volume 1 µL, injector temperature 230 °C, detector temperature 320 °C, N_2_ as carrier gas. Retention times: **1a**, 9.57 min and 9.78 min; **3a**, 9.21 min.

## 4.3 NMR

The residual solvent signal of CDCl₃ was used as the reference for chemical-shift calibration.

**Figure S19**. ^1^H NMR (400 MHz, CDCl_3_) of **2a**, isolated from the 100 mL scale biotransformation.

**Figure S20**. ^13^C NMR (400 MHz, CDCl_3_) of **2a**, isolated from the 100 mL scale biotransformation.

**Figure S21**. ^1^H NMR (400 MHz, CDCl_3_) of **2b**.

**Figure S22**.^13^C NMR (400 MHz, CDCl_3_) of **2b**.

**Figure S23**. ^1^H NMR (400 MHz, CDCl_3_) of **2c**.

**Figure S24**. ^13^C NMR (400 MHz, CDCl_3_) of **2c**.

**Figure S25**. ^1^H NMR (400 MHz, CDCl_3_) of **2d**.

**Figure S26**. ^13^C NMR (400 MHz, CDCl_3_) of **2d**.

**Figure S27**. ^1^H NMR (400 MHz, CDCl_3_) of **2e**, isolated from a 20 mL scale biotransformation.

**Figure S28**. ^13^C NMR (400 MHz, CDCl_3_) of **2e**, isolated from a 20 mL scale biotransformation.

**Figure S29**. ^1^H NMR (400 MHz, CDCl_3_) of **2f**.

**Figure S30**. ^13^C NMR (400 MHz, CDCl_3_) of **2f**.

## 4.4 Representative chromatograms

**Figure S31.** Representative HPLC chromatograms for quantification of **2a**. (**A**) Reference chromatogram of **2a** standard. (**B**) Reaction sample from chemoenzymatic synthesis.

**Figure S32.** Representative HPLC chromatograms for quantification of **2b**. (**A**) Reference chromatogram of **2b** standard. (**B**) Reaction sample from chemoenzymatic synthesis.

**Figure S33.** Representative HPLC chromatograms for quantification of **2c**. (**A**) Reference chromatogram of **2c** standard. (**B**) Reaction sample from chemoenzymatic synthesis.

**Figure S34.** Representative HPLC chromatogram for quantification of **2d**. (**A**) Reference chromatogram of **2d** standard. (**B**) Reaction sample from chemoenzymatic synthesis.

**Figure S35.** Representative HPLC chromatogram for quantification **2e**. (**A**) Reference chromatogram of **2e** standard. (**B**) Reaction sample from chemoenzymatic synthesis.

**Figure S36.** Representative HPLC chromatogram for quantification of **2f**. (**A**) Reference chromatogram of **2f** standard. (**B**) Reaction sample from chemoenzymatic synthesis.

**Figure S37.** Representative HPLC chromatogram for quantification **2g**. (**A**) Reference chromatogram of **2g** standard. (**B**) Reaction sample from chemoenzymatic synthesis.

**Figure S38.** Representative HPLC chromatogram for detection of **2h**. (**A**) Reaction sample from chemoenzymatic synthesis. (**B**) Mass spectrum of the corresponding peak (m/z 150 [M+1], 191 [M+42]).

**Figure S39.** Representative HPLC chromatogram for quantification of **2i**. (**A**) Reaction sample from chemoenzymatic synthesis. (**B**) Mass spectrum of the corresponding peak (m/z 152 [M+1], 193 [M+42]).

**Figure S40**. Representative HPLC chromatograms for detection of aminoacetone and 1-aminopropan-2-ol. (**A**) Benzoylated aminoacetone reference. (**B**) Benzoylated 1-aminopropan-2-ol reference. (**C**) Benzoylated reaction sample of the biotransformation towards **2a** using whole-cell *Ec*ThrDH/ADH-A. (**D**) Benzoylated reaction sample of the biotransformation towards **2a** using whole-cell *Ec*ThrDH. Additional signals observed in the reaction samples prevented reliable quantitative determination of the two compounds, but the peak identities were confirmed by MS analysis.

**Figure S41.** Representative GC chromatogram for detection of **1a** and quantification of **3a** as trimethylsilyl esters. (**A**) Reference chromatogram of **1a** standard. The two peaks correspond to the stereoisomers (E and Z), originating from the keto-enol equilibrium of the *β*-keto ester. (**B**) Reference chromatogram of **3a** standard. (**C**) ADH-A catalyzed reduction of **1a** to **3a** coupled to the chemoenzymatic cascade for pyrrole synthesis.

## 4.5 Calibration curves

Pyrrole calibration samples were prepared analogously to the biotransformation reactions; however, methanol (MeOH) was used instead of buffer, to address solubility limitations. Samples in which product concentrations exceeded 50 mM were further diluted with MeOH to fall within the calibration range.

**Figure S42.** Calibration curves for the quantification of pyrroles **2a-g**, 2,5-dimethylpyrazine (2,5-DMP) and **3a**. The corresponding line equations and R^2^ values were generated by a simple linear regression.

## 4.6 DNA sequences

### 4.6.1 *Cn*ThrDH

ATGCATCATCATCACCATCATAGCAGCGGCGAAGCGGGCAAACCGAAAATTCTGATTGTGGGCGCGAACGGCCAGATTGGCAGCGAACTGGCGCTGGCGCTGGCGGAACGCTATGGCCGCACCAACGTGATTACCAGCGATGTGGTGCCGACCGGCCGCCATGTGCATCTGACCCATGAAATGCTGAACGCGACCGATCGCGGCGAACTGGCGACCGTGGTGGAACGCCATGGCATTACCCAGGTGTATCTGCTGGCGGCGGCGCTGAGCGCGACCGGCGAAAAAGCGCCGCAGTGGGCGTGGAACCTGAACATGACCAGCCTGCTGAACGTGCTGGAACTGGCGCGCCAGACCGGCCTGGAACGCGTGTTTTGGCCGAGCAGCATTGCGGCGTTTGGCCCGACCACCCCGGCGGGCCAGACCCCGCAGAAAACCGTGATGGAACCGACCACCGTGTATGGCATTAGCAAACAGGCGGGCGAAGGCTGGTGCCGCTGGTATCATGCGAACCATGGCGTGGATGTGCGCAGCGTGCGCTATCCGGGCCTGATTAGCCATAAAACCCCGCCGGGCGGCGGCACCACCGATTATGCGGTGGATATTTTTCATGCGGCGGTGACCGGCGAACCGTATACCTGCTTTCTGAAAGAAGATGAAGCGCTGCCGATGATGTATATGCCGGATGCGATTCGCGCGACCATTGAACTGATGGAAGCGCCGGCGGATAAACTGAGCGAACGCGGCAGCTATAACATTGCGGGCATGAGCTTTACCCCGGCGCAGATTGCGGCGGCGATTCGCGAACAGGTGCCGGGCTTTCAGATTCGCTATGAACCGGATTATCGCCAGGCGATTGCGCAGGGCTGGCCGGATAGCATTGATGATAGCGTGGCGCGCGCGGATTGGGGCTGGAAAGCGCAGTATGGCCTGAAAGAAATGGTGGCGGATATGCTGGCGAACCTGAAAGCGACCCTGGCGGGCTAA

### 4.6.2 *Bs*ThrDH

ATGCATCATCACCACCACCATGGTTCCGGTATGAAGAAAATTCTGGTTACAGGCAGCCTGGGGCAGATAGGCTCGGAATTGGTCATGAAGTTACGCGACGTGTACGGGGCGAGTAATGTTATCGCGACTGATATTCGTGAAACCGATTCAGAAGTAGTGACCAGCGGACCGTTTGAAACCCTTGATGTCACCGATGGTCAGAAACTCCATGACATTGCAAAACGCAATGAAGTTGACACGATTATTCACCTGGCCGCGCTTCTGAGCGCGACGGCGGAGAAAAATCCTCTGTTCGCGTGGAATCTGAACATGGGTGGCCTAGTGAATGCGTTAGAAGCCGCACGGGAACTGAACTGTAAGTTCTTTACCCCGAGCTCGATAGGTGCTTTTGGCCCTTCGACGCCGAAAGACAACACTCCGCAAGATACCATTCAACGACCTACAACTATGTACGGCGTAAACAAAGTGGCGGGCGAACTCCTGTGCGATTACTATCATCAGAAATTCGGCGTAGATACCCGTGGCGTGCGCTTCCCGGGCTTAATTAGCTACGTTGCACCGCCAGGTGGAGGTACCACAGATTATGCGGTCGAAATTTACTATGAGGCAATCAAAAAAGGAGCCTACACCTCCTACATTGCAGAAGGCACCTATATGGACATGATGTATATGCCCGACGCCCTCCAGGCGATCATCTCATTGATGGAAGCGGATCCAAGTAAACTGGTGCATCGTAACGCTTTTAACATTACCGCTATGTCTTTTGAGCCCGAGCAGATTGCCGCGTCTATCCGCAAACACATCCCGACATTCACGATGGATTATGCCGTTGATCCGGCACGTCAGACGATTGCAGATAGCTGGCCGAATTCCATCGACGCCACCGCTGCGATGAAAGAATGGGGTTTTAAAGCCGAATATGACTTGGATAAGATGACTACCGATATGCTTGCCAAATTAAAGAAGAAACTGACGGCCGAGCTGGTGATGAACTAA

### 4.6.3 *Ec*ThrDH

ATGCATCATCACCACCACCATGGTTCCGGTATGAAAGCGTTATCCAAACTGAAAGCGGAAGAGGGCATCTGGATGACCGACGTTCCTGTACCGGAACTCGGGCATAACGATCTGCTGATTAAAATCCGTAAAACAGCCATCTGCGGGACTGACGTTCACATCTATAACTGGGATGAGTGGTCGCAAAAAACCATCCCGGTGCCGATGGTCGTGGGCCATGAATATGTCGGTGAAGTGGTAGGTATTGGTCAGGAAGTGAAAGGCTTCAAGATCGGCGATCGCGTTTCTGGCGAAGGCCATATCACCTGTGGTCATTGCCGCAACTGTCGTGGTGGTCGTACCCATTTGTGCCGCAACACGATAGGCGTTGGTGTTAATCGCCCGGGCTGCTTTGCCGAATATCTGGTGATCCCGGCATTCAACGCCTTCAAAATCCCCGACAATATTTCCGATGACTTAGCCGCAATTTTTGATCCCTTCGGTAACGCCGTGCATACCGCGCTGTCGTTTGATCTGGTGGGCGAAGATGTGCTGGTTTCTGGTGCAGGCCCGATTGGTATTATGGCAGCGGCGGTGGCGAAACACGTTGGTGCACGCAATGTGGTGATCACTGATGTTAACGAATACCGCCTTGAGCTGGCGCGTAAAATGGGTATCACCCGTGCGGTTAACGTCGCCAAAGAAAATCTCAATGACGTGATGGCGGAGTTAGGCATGACCGAAGGTTTTGATGTCGGTCTGGAAATGTCCGGTGCGCCGCCAGCGTTTCGTACCATGCTTGACACCATGAATCACGGCGGCCGTATTGCGATGCTGGGTATTCCGCCGTCTGATATGTCTATCGACTGGACCAAAGTGATCTTTAAAGGCTTGTTCATTAAAGGTATTTACGGTCGTGAGATGTTTGAAACCTGGTACAAGATGGCGGCGCTGATTCAGTCTGGCCTCGATCTTTCGCCGATCATTACCCATCGTTTCTCTATCGATGATTTCCAGAAGGGCTTTGACGCTATGCGTTCGGGCCAGTCCGGGAAAGTTATTCTGAGCTGGGATTAA

### 4.6.4 *Lok*ThrDH

ATGCATCATCACCACCACCATGGTTCCGGTATGACCAACCAAATGAAAGCCCTTGAAAAGAGTAGGCCCGAACCGGGATTATGGATGGTAAACGCACCGGTTCCCGAAATTGGACCAGATGACGTATTGATTAAAGTTAACAAAACCGGAATTTGTGGTACAGATATTCATATATGGAATTGGGATGACTGGGCGGCTCGGACTGTACCTGTTCCGCTGATTACCGGTCACGAATTCGCGGGTGAAATTGTTGAAATCGGACGTAATGTGGAAGGGCTGGAAATCGGGCAGCGCTGCTCTGGTGAGGGCCATCTAATTGGTAAAAAATCCCGCCAGGCGCGCGCCGGTAAATTTCATTTAGACCCGGCCACGCGAGGCATCGGTGTGAACGAACAGGGCGCCTTTGCCCAGTATCTGCGTCTCCCTGCATTCAACGTGGTTCCGCTGCCGGACGCGATTGACGATGAAATCGGCGCGATCTTGGACCCTCTCGGCAACGCTGTGCACACCGCTCTGAGCTTTGATCTGGTGGGCGAGGATGTCCTCATTACTGGCGCGGGCCCAATCGGTATTATGGCCGCGGCGGTAGCACGTCACGTGGGCGCCCGTCATGTGGTCATCACTGATGTCAACCAAGACCGTCTTGATCTGGCCGCGCGTGTGGCAGATGTTGTTCCAGTCAATGTCGCGAAGCAGGATCTGCGCGATGTCGAAGGCGCACTGCGCATGACGGAGGGTTTCGATGTTGGTCTGGAGATGAGCGGCAATCAGCAAGCCTTAGACCAGATGGTGGAAAATCTGGTGATGGGTGGCCGTATTGCGTTACTGGGCATCCCGCCGGGAAAATCACCGGTGGATTGGAGCCGAATAGTATTTAAGGCAATTACAATCAAGGGGGTGTACGGCCGCGAAATTTTTGAGACCTGGTATAAAATGATTGCAATGCTTGAAAATGGCTTGAAAGTACGCGACGTGATTACCCACCGCTTTAAAGCCGATGATTTCCGCACGGGGTTCGAGACGATGAAAGGGGGCGCGTCGGGCAAAGTTGTGCTGGATTGGACCTAA

### 4.6.5 ADH-A

ATGAAAGCGGTGCAGTATACCGAAATTGGCAGCGAACCGGTGGTGGTGGATATTCCGACCCCGACCCCGGGCCCGGGCGAAATTCTGCTGAAAGTGACCGCGGCGGGCCTGTGCCACAGCGATATTTTTGTGATGGATATGCCGGCGGCGCAGTATGCGTATGGCCTGCCGCTGACCCTGGGCCACGAAGGCGTGGGCACCGTGGCGGAACTGGGCGAAGGCGTGACCGGCTTTGGCGTGGGCGATGCGGTGGCGGTGTATGGCCCGTGGGGCTGCGGCGCGTGCCACGCGTGCGCGCGCGGCCGCGAAAACTATTGCACCCGCGCGGCGGATCTGGGCATTACCCCGCCGGGCCTGGGCAGCCCGGGCAGCATGGCGGAATATATGATTGTGGATAGCGCGCGCCACCTGGTGCCGATTGGCGATCTGGATCCGGTGGCGGCGGCGCCGCTGACCGATGCGGGCCTGACCCCGTACCACGCGATTAGCCGCGTGCTGCCGCTGCTGGGCCCGGGCAGCACCGCGGTGGTGATTGGCGTGGGCGGCCTGGGCCACGTGGGCATCCAGATTCTGCGCGCGGTGAGCGCGGCGCGCGTGATTGCGGTGGATCTGGATGATGATCGCCTGGCGTTGGCGTGCAAAGTGGGCGCGGATGCGGCGGTGAAAAGCGGCGCGGGCGCGGCGGATGCGATTCGCGAACTGACCGGCGGCCAGGGCGCGACCGCGGTGTTTGATTTTGTGGGCGCGCAGAGCACCATTGATACCGCGCAGCAGGTGGTGGCGGTGGATGGCCACATTAGCGTGGTGGGTATTCACGCGGGCGCGCACGCGAAAGTGGGCTTTTTTATGATTCCGTTTGGCGCGAGCGTGGTGACCCCGTATTGGGGCACCCGCAGCGAACTGATGGAAGTGGTGGCGCTGGCGCGCGCGGGCCGCCTGGATATTCACACCGAAACCTTTACCCTGGATGAAGGCCCGGCGGCGTATCGCCGCCTGCGCGAAGGCAGCATTCGCGGCCGCGGCGTGGTGGTGCCGTAA

### 4.6.6 ADH-hT

ATGAAAGCGGCGGTGGTGGAACAGTTTAAAGAACCGCTGAAAATTAAAGAAGTGGAAAAACCGACCATTAGCTATGGCGAAGTGCTGGTGCGCATTAAAGCGTGCGGCGTGTGCCATACCGATCTGCATGCGGCGCATGGCGATTGGCCGGTGAAACCGAAACTGCCGCTGATTCCGGGCCATGAAGGCGTGGGCATTGTGGAAGAAGTGGGCCCGGGCGTGACCCATCTGAAAGTGGGCGATCGCGTGGGCATTCCGTGGCTGTATAGCGCGTGCGGCCATTGCGATTATTGCCTGAGCGGCCAGGAAACCCTGTGCGAACATCAGAAAAACGCGGGCTATAGCGTGGATGGCGGCTATGCGGAATATTGCCGCGCGGCGGCGGATTATGTGGTGAAAATTCCGGATAACCTGAGCTTTGAAGAAGCGGCGCCGATTTTTTGCGCGGGCGTGACCACCTATAAAGCGCTGAAAGTGACCGGCGCGAAACCGGGCGAATGGGTGGCGATTTATGGCATTGGCGGCCTGGGCCATGTGGCGGTGCAGTATGCGAAAGCGATGGGCCTGAACGTGGTGGCGGTGGATATTGGCGATGAAAAACTGGAACTGGCGAAAGAACTGGGCGCGGATCTGGTGGTGAACCCGCTGAAAGAAGATGCGGCGAAATTTATGAAAGAAAAAGTGGGCGGCGTGCATGCGGCGGTGGTGACCGCGGTGAGCAAACCGGCGTTTCAGAGCGCGTATAACAGCATTCGCCGCGGCGGCGCGTGCGTGCTGGTGGGCCTGCCGCCGGAAGAAATGCCGATTCCGATTTTTGATACCGTGCTGAACGGCATTAAAATTATTGGCAGCATTGTGGGCACCCGCAAAGATCTGCAGGAAGCGCTGCAGTTTGCGGCGGAAGGCAAAGTGAAAACCATTATTGAAGTGCAGCCGCTGGAAAAAATTAACGAAGTGTTTGATCGCATGCTGAAAGGCCAGATTAACGGCCGCGTGGTGCTGACCCTGGAAGATAAATAA

# 5 References

[1]	T. Motoyama, S. Nakano, F. Hasebe, R. Miyata, S. Kumazawa, N. Miyoshi, S. Ito, “Chemoenzymatic Synthesis of 3-ethyl-2,5-dimethylpyrazine by l-Threonine 3-Dehydrogenase and 2-Amino-3-Ketobutyrate CoA Ligase/l-Threonine Aldolase” *Commun. Chem.* **2021**, *4*, 108.

[2]	J. Xu, A. P. Green, N. J. Turner, “Chemo-Enzymatic Synthesis of Pyrazines and Pyrroles” *Angew. Chem. Int. Ed.* **2018**, *57*, 16760–16763.

[3]	S. Nakano, S. Okazaki, H. Tokiwa, Y. Asano, “Binding of NAD^+^ and l-Threonine Induces Stepwise Structural and Flexibility Changes in *Cupriavidus necator* l-Threonine Dehydrogenase” *J. Biol. Chem.* **2014**, *289*, 10445–10454.

[4]	K. Noda, M. Igarashi, Y. Morimitsu, S. Tsutsuura, M. Murata, “2,5-Dimethylpyrazine and 2,3,5-Trimethylpyrazine in Natto are Formed Metabolically from Threonine through Aminoacetone by *Bacillus natto* During the Fermentation” *Food Sci. Technol. Res.* **2025**, *31*, 427–439.

[5]	G. Kłosowski, D. Mikulski, K. Pielech-Przybylska, “Pyrazines Biosynthesis by *Bacillus* Strains Isolated from Natto Fermented Soybean” *Biomolecules* **2021**, *11*, 1736.

[6]	S. A. Boylan, E. E. Dekker, “l-Threonine Dehydrogenase: Purification and Properties of the Homogeneous Enzyme from *Escherichia coli* K-12” *J. Biol. Chem.* **1981**, *256*, 1809–1815.

[7]	A. R. Johnson, Y.-W. Chen, E. E. Dekker, “Investigation of a Catalytic Zinc Binding Site in *Escherichia coli* l-Threonine Dehydrogenase by Site-Directed Mutagenesis of Cysteine-38” *Arch. Biochem. Biophys.* **1998**, *358*, 211–221.

[8]	J. Feng, R. Li, S. Zhang, Y. Bu, Y. Chen, Y. Cui, B. Lin, Y. Chen, Y. Tao, B. Wu, “Bioretrosynthesis of Functionalized *N*‐Heterocycles from Glucose via One‐Pot Tandem Collaborations of Designed Microbes” *Adv. Sci.* **2020**, *7*, 2001188.

[9]	J. S. Dickschat, I. Wagner-Döbler, S. Schulz, “The Chafer Pheromone Buibuilactone and Ant Pyrazines are also Produced by Marine Bacteria” *J. Chem. Ecol.* **2005**, *31*, 925–947.

[10]	W. Stampfer, B. Kosjek, K. Faber, W. Kroutil, “Biocatalytic Asymmetric Hydrogen Transfer Employing *Rhodococcus ruber* DSM 44541” *J. Org. Chem.* **2003**, *68*, 402–406.

[11]	B. Kosjek, W. Stampfer, M. Pogorevc, W. Goessler, K. Faber, W. Kroutil, “Purification and Characterization of a Chemotolerant Alcohol Dehydrogenase Applicable to Coupled Redox Reactions” *Biotechnol. Bioeng.* **2004**, *86*, 55–62.

[12]	M. Karabec, A. Łyskowski, K. C. Tauber, G. Steinkellner, W. Kroutil, G. Grogan, K. Gruber, “Structural Insights into Substrate Specificity and Solvent Tolerance in Alcohol Dehydrogenase ADH-‘A’ from *Rhodococcus ruber* DSM 44541” *Chem. Commun.* **2010**, *46*, 6314–6316.

[13]	A. Guagliardi, M. Martino, I. Iaccarino, M. D. Rosa, M. Rossi, S. Bartolucci, “Purification and Characterization of the Alcohol Dehydrogenase from a Novel Strain of *Bacillus stearothermophilus* Growing at 70°C” *Int. J. Biochem. Cell Biol.* **1996**, *28*, 239–246.

[14]	D.-Y. Zheng, T. Zhang, R. Bai, M. Li, R. Wang, Y. Gu, “Synthesis of Unsymmetrical NH-pyrroles from Biomass Feedstock in the Confined Space of Metal–Organic Frameworks” *Green Chem.* **2023**, *25*, 915–921.

[15]	L. Sun, C. Liang, S. Shirazian, Y. Zhou, T. Miller, J. Cui, J. Y. Fukuda, J.-Y. Chu, A. Nematalla, X. Wang, H. Chen, A. Sistla, T. C. Luu, F. Tang, J. Wei, C. Tang, “Discovery of 5-[5-fluoro-2-oxo-1,2-dihydroindol-(3*Z*)-ylidenemethyl]-2,4-dimethyl-1*H*-pyrrole-3-carboxylic acid (2-diethylaminoethyl)amide, a Novel Tyrosine Kinase Inhibitor Targeting Vascular Endothelial and Platelet-Derived Growth Factor Receptor Tyrosine Kinase” *J. Med. Chem.* **2003**, *46*, 1116–1119.

[16]	P. Clezy, B. Ravi, L. Thuc, “The Chemistry of Pyrrolic Compounds. LIX. The Oxidative Cyclization of Bilenes-b Substituted by Ethyl or Propyl Groups at a Terminal Site” *Aust. J. Chem.* **1986**, *39*, 419–431.
